# Supplementary material for: Mechanism of Crosstalk between the LSD1 Demethylase and HDAC1 Deacetylase in the CoREST Complex
Source: Cell Rep. 2020 Feb 25;30(8):2699–2711.e8. doi: 10.1016/j.celrep.2020.01.091 (PMC7043024; doi:10.1016/j.celrep.2020.01.091)
Supplement: Document S1. Figures S1–S7 and Tables S1–S7 [file mmc1.pdf]

Cell Reports, Volume *30*

Supplemental Information

Mechanism of Crosstalk between the LSD1 Demethylase and HDAC1 Deacetylase in the CoREST Complex

Yun Song, Lisbeth Dagil, Louise Fairall, Naomi Robertson, Mingxuan Wu, T.J. Ragan, Christos G. Savva, Almutasem Saleh, Nobuhiro Morone, Micha B.A. Kunze, Andrew G. Jamieson, Philip A. Cole, D. Flemming Hansen, and John W.R. Schwabe

Supplementary Figures and Tables:

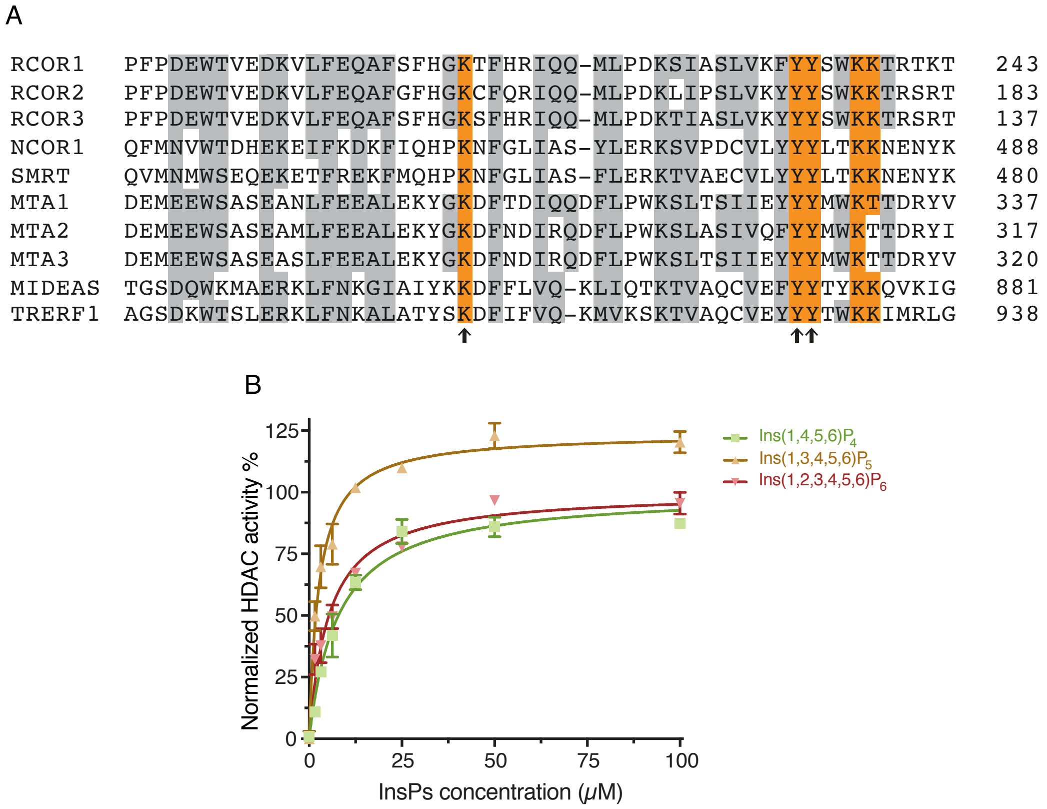

Figure S1. Related to Figure 1. Regulation of the HDAC activity of the CoREST complexes by inositol phosphates.

(A) Sequence alignment of the N-terminal SANT domain of the RCOR1 protein to several other SANT domain containing proteins. Residues that mediate the interaction with Ins(1,4,5,6)P_4 from SMRT SANT domain are highlighted in orange. Black arrows indicate the residues that are essential for the inositol phosphate binding and complex activation.

(B) Activation of HDAC1 in the LSD1:RCOR1:HDAC1 complex by different inositol phosphates. Data were normalized to the HDAC activity in the present of 200 μM of Ins(1,4,5,6)P_4. Error bars indicate the SEM (n=9).

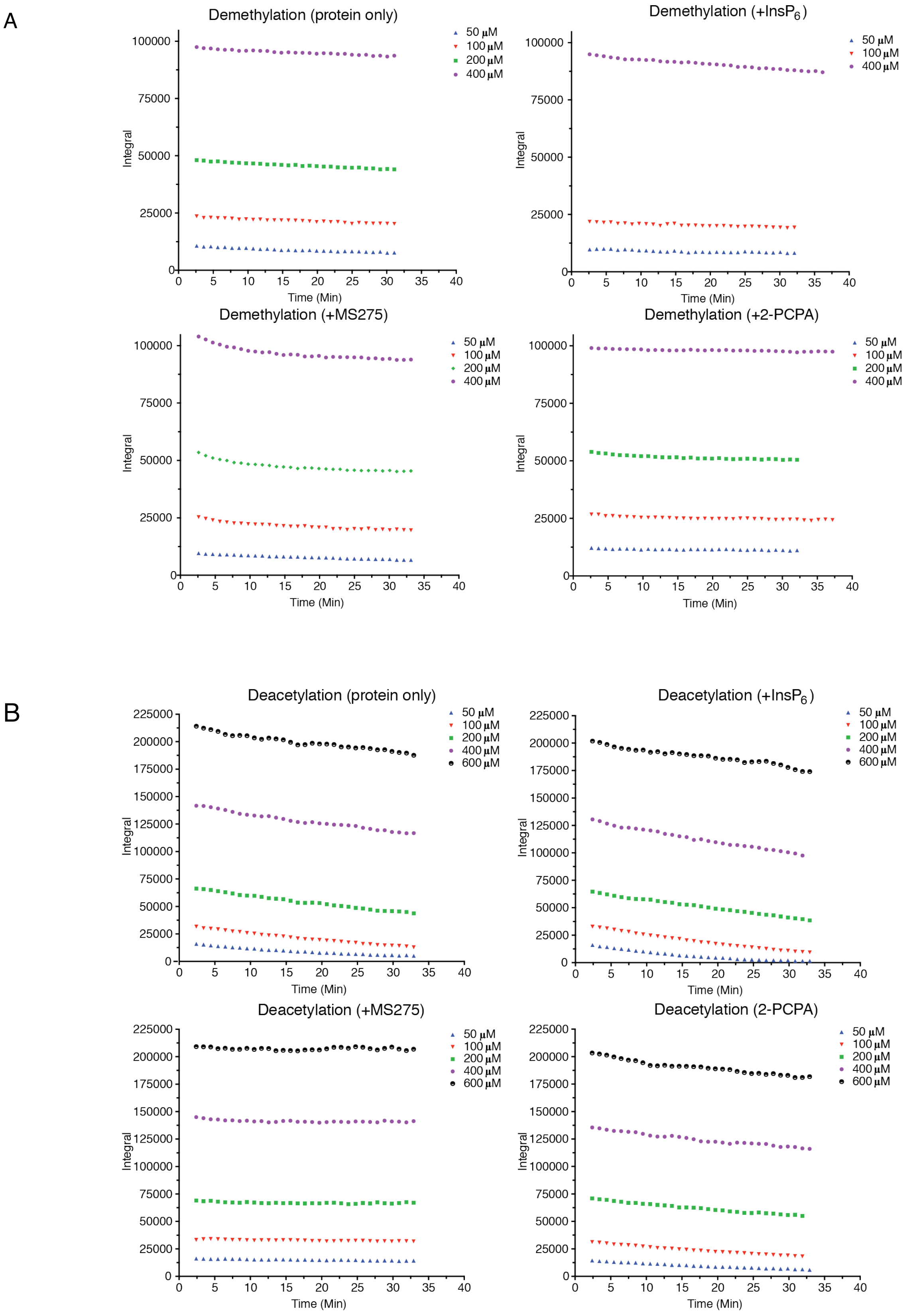

Figure S2. Related to Figure 2.

(A) Time-course data obtained by integrating the substrate peak of monomethylated K4 of the H3 K4meK9 substrate. 200 nM of CoREST ternary complex was used in the experiments.

(B) Time-course data obtained by integrating the substrate peak of acetylated K9 of the H3 K4K9ac substrate. 50 nM of CoREST ternary complex was used in the experiments.

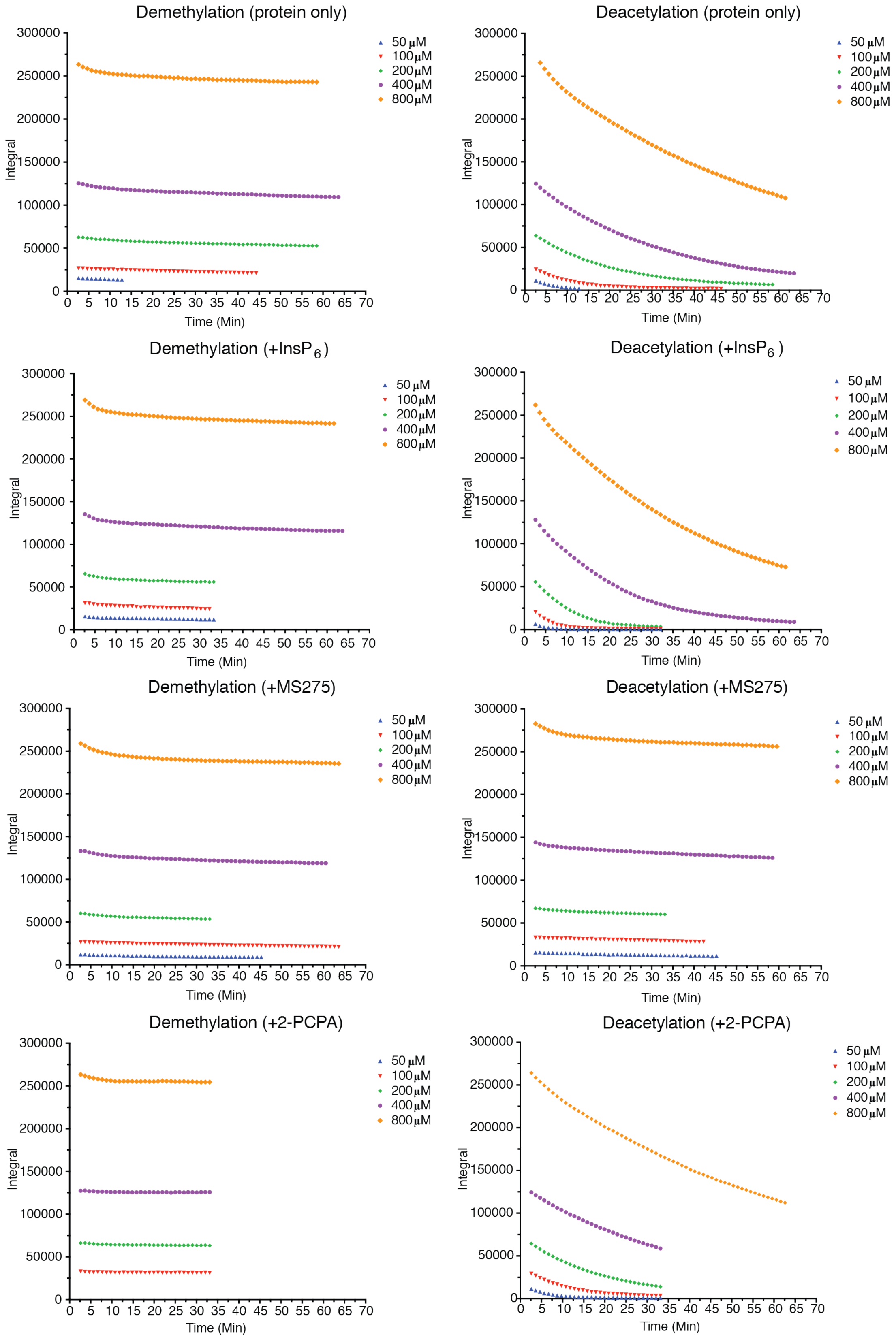

Figure S3. Related to Figure 2. Time-course data obtained by integrating the substrate peak of monomethylated K4 and acetylated K9 of the H3 K4meK9ac substrate. 200 nM of CoREST ternary complex was used in the experiments.

A

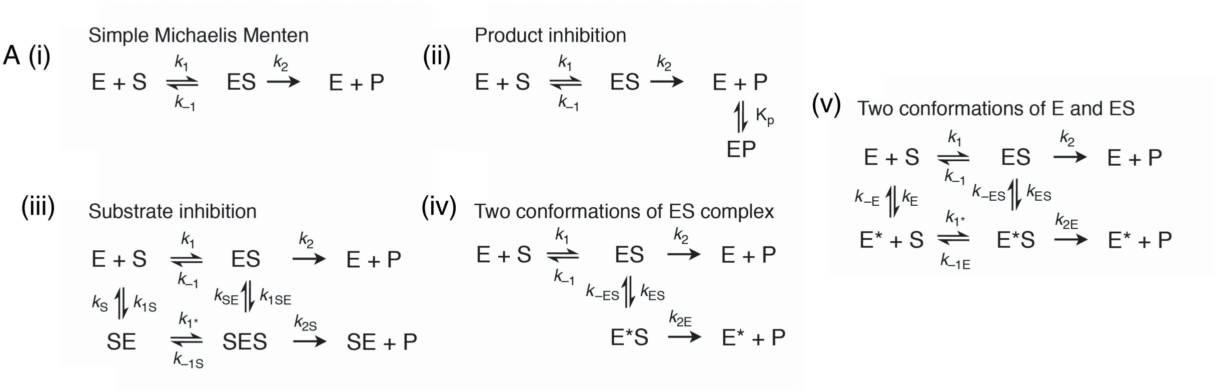

B

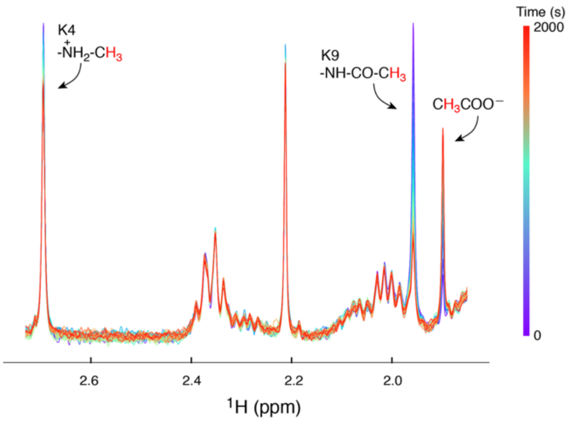

C

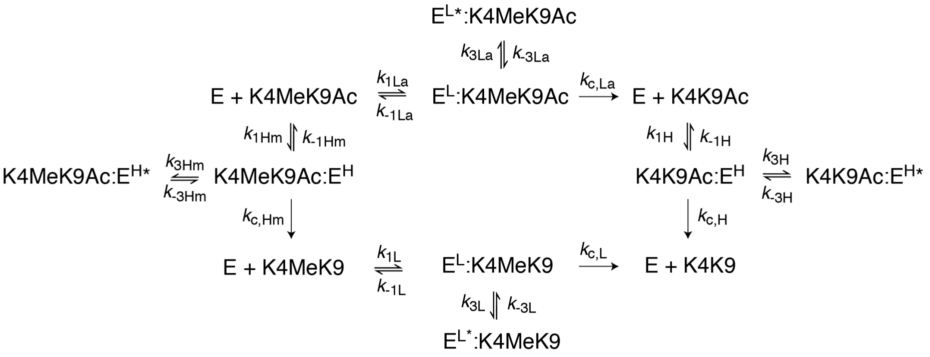

Figure S4. Related to Figure 2.

(A) Models considered for the demethylation of H3 K4meK9 and the deacetylation of H3 K4K9ac: A simple (i) Michaelis-Menten reaction scheme; (ii) Michaelis-Menten reaction with product-inhibition. (The equilibrium between E+P and EP is described by the equilibrium constant, K_p, and is thus assumed to be reached instantaneous); (iii) Michaelis-Menten with generalised substrate inhibition of both the free enzyme, E, and the enzyme-substrate complex, ES; (iv) Reaction scheme where the enzyme-substrate complex can exist in two conformations; (v) Reaction scheme where both the free enzyme, E, and the enzyme-substrate complex, ES, can exist in two conformations. For all reactions k_1 = 0.25×10^6 $M^{-1}s^{-1}$ corresponding to near diffusion limit on-rate. Small changes in k_1 did not change the obtained χ^2. For the reaction schemes in (iii) and (v) $k_{1^*} = k_1$. Also, there are only 7 fitting parameters in scheme (iii) and (v), despite 8 shown, due to the cyclic nature of the schemes. Thus, in (v), k_{-1E}, can be calculated from k_{1^*}, k_{-E}, k_E, k_1, k_{-1}, k_{-ES}, k_{ES}.

(B) Series of ^1H NMR spectra showing the demethylation and deacetylation of the substrate H3K4meK9ac (50 μM) by CoREST (200 nM).

(C) Reaction scheme used for the analysis of combined demethylation and deacetylation of the doubly modified histone H3 K4meK9ac substrate. All second-order association rates, k_{1Hm}, k_{1H}, k_{1La}, k_{1L} were fixed to $2\cdot10^5$ $M^{-1}s^{-1}$ as discussed in the main text

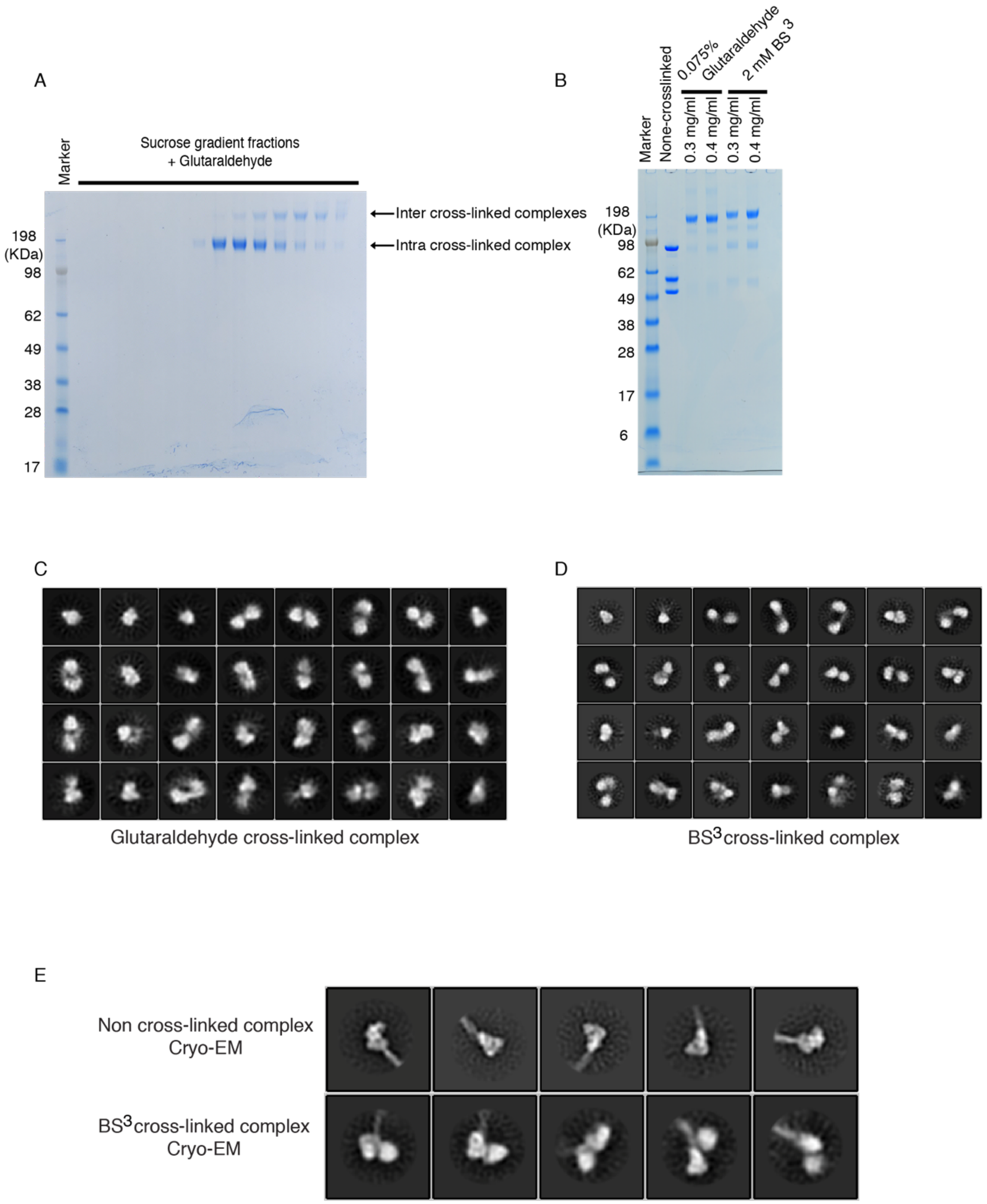

Figure S5. Related to Figure 4. Cryo-electron microscopy of the CoREST complex.

(A) Purification and crosslinking of the CoREST complex using a 5-25% sucrose (+0.1% glutaraldehyde) gradient. NuPAGE gel analysis of fractions 1-14 from sucrose gradient.

(B) Crosslinking the CoREST complex with 0.075% glutaraldehyde and 2 mM BS^3 in a microfuge tube for 5 minutes at room temperature.

(C) Selected 2D-classes of the CoREST complex cross-linked with glutaraldehyde.

(D) Selected 2D-classes of the CoREST complex cross-linked with BS^3.

(E) Selected 2D-class averages of the non cross-linked CoREST complex and BS^3 cross-linked CoREST complex in Cryo-EM.

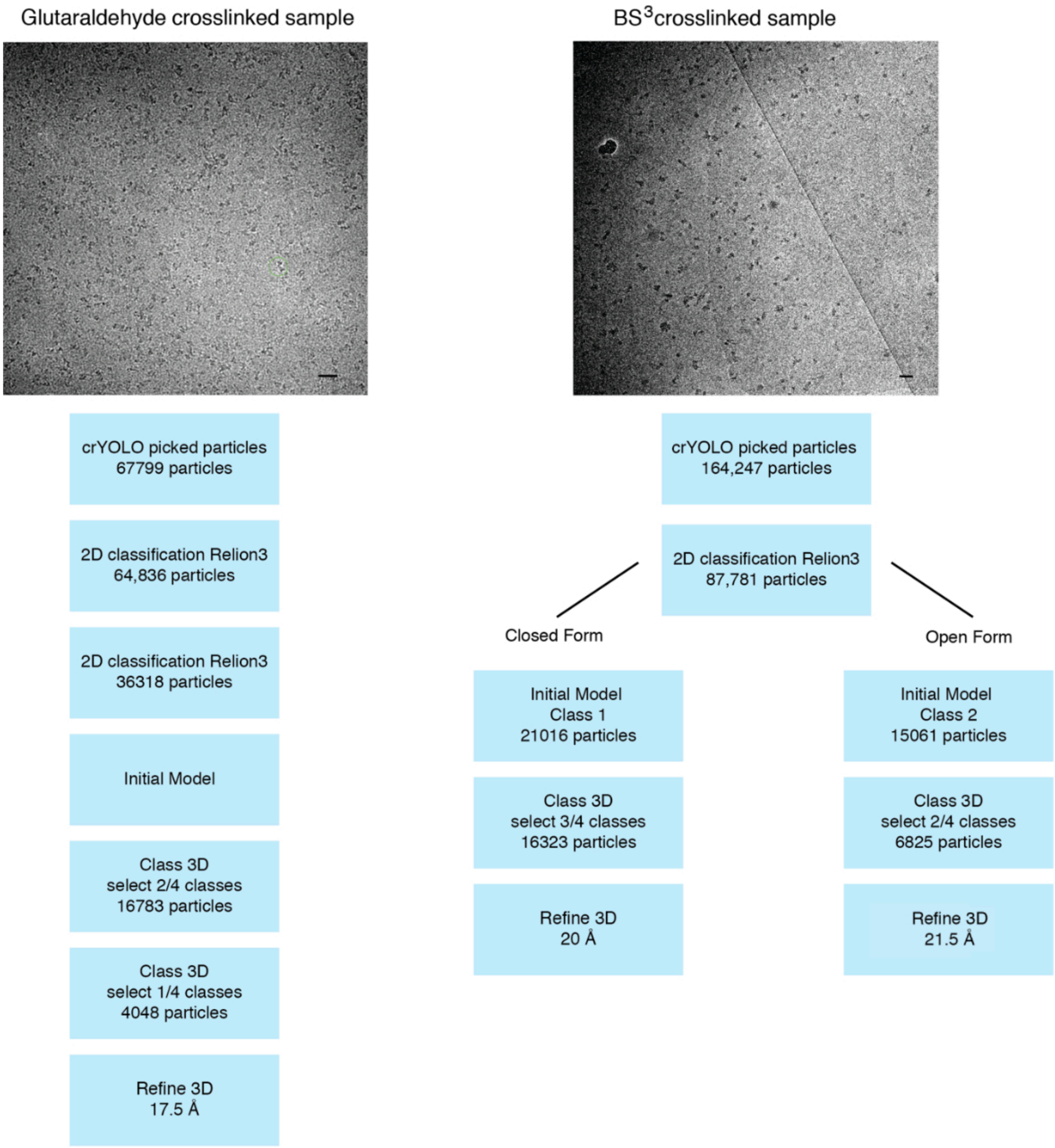

Figure S6. Related to Figure 4. Typical micrographs and flow chart for the Relion 3.0 processing of the cryo-EM datasets for the glutaraldehyde and BS^3 crosslinked CoREST samples. The scale bar in the micrographs is 20 nm.

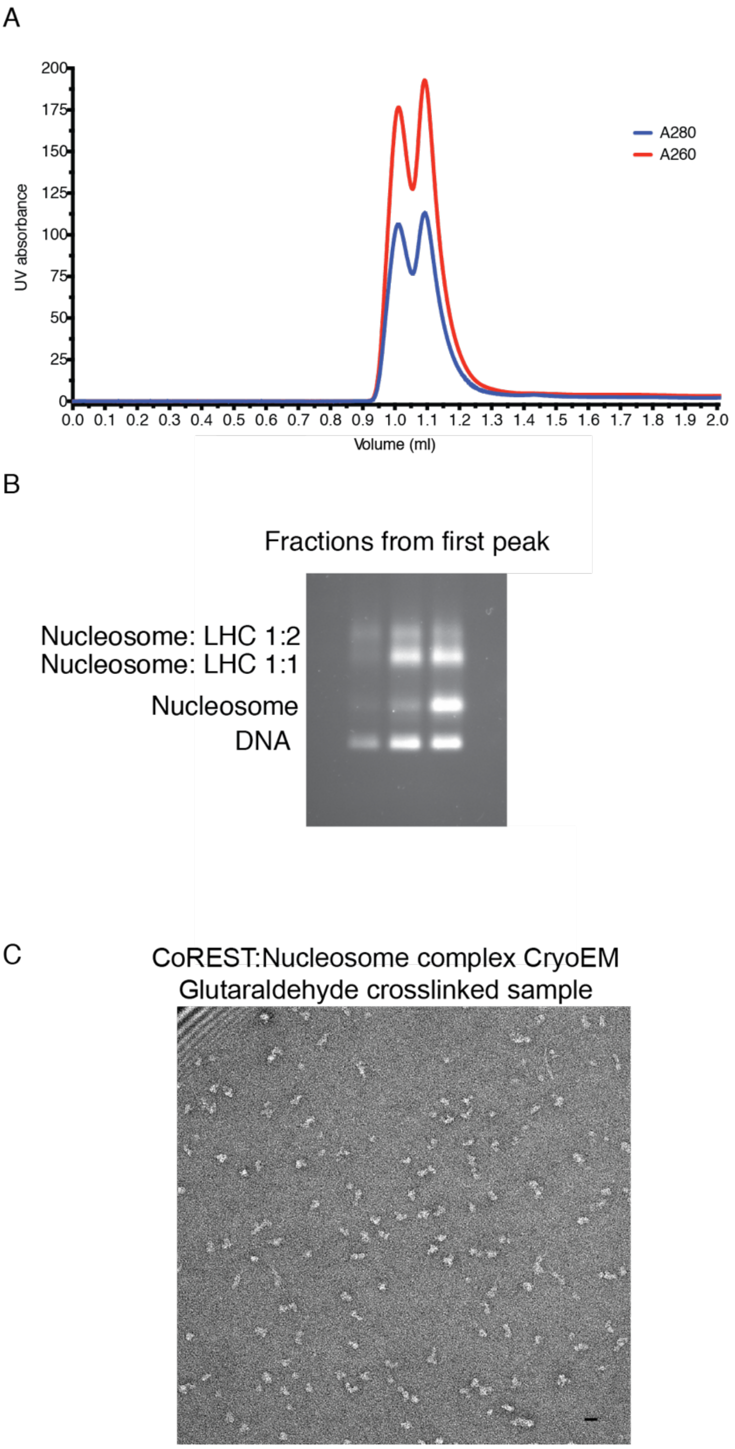

Figure S7. Related to Figure 5. Purification and negative stain microscopy of the nucleosome bound CoREST complex.

(A) CoREST complex was mixed with nucleosome in a molar ratio of 3:1 and then purified through a 2.4 ml Superdex 200 (3.2/300) column with a fraction size of 50 μl.

(B) The fractions from the first peak (0.9ml - 1.05 ml) were analysed on a 0.7% agarose gel in 0.5x TB buffer. The gel was stained with ethidium bromide and visualized by UV.

(C) Typical electron micrograph of the negatively stained CoREST:nucleosome complex. The scale bar is 20 nm.

Table S1. Related to Figure 2. Characterisation data for purified peptides.

Peptide	Calculated MW [M+H]+ (Da)	Run time (min)	ES+ peaks (m/z)	Purity (%)	Yield (%)
**H-ARTK(Me)QTARKSTGGKAPRKQLA-NH_2 (H3(1-21)K4me)**	2267.3530	11.797[a] 13.223[b]	1134.5880 $[M+2H]^{2+}$ 756.7203 $[M+3H]^{3+}$ 567.8007 $[M+4H]^{4+}$ 454.4461 $[M+5H]^{5+}$ 378.6996 $[M+6H]^{6+}$	>99	45
**H-ARTKQTARK(Ac)STGGKAPRKQLA-NH_2 (H3(1-21)K9Ac)**	2295.3441	11.783[a] 13.193[b]	1148.6685 $[M+2H]^{2+}$ 766.1127 $[M+3H]^{3+}$ 574.8332 $[M+4H]^{4+}$ 460.0672 $[M+5H]^{5+}$ 384.1527 $[M+6H]^{6+}$	>99	36
**H-ARTK(Me)QTARK(Ac)STGGKAPRKQLA-NH_2 (H3(1-21)K4meK9ac)**	2309.3636	11.823[a] 13.300[b]	1155.7358 $[M+2H]^{2+}$ 770.8013 $[M+3H]^{3+}$ 578.3477 $[M+4H]^{4+}$ 462.8733 $[M+5H]^{5+}$ 385.7206 $[M+6H]^{6+}$	>99	23

Analytical reverse-phase HPLC using a Dionex Ultimate 3000 system with a Phenomenex Aeris 5 μm Peptide XB-C18 100 Å packed column with dimensions of 150 x 4.6 mm, using the following gradients: (a) 5-100% MeCN/H_2O (0.1% TFA), 15 min gradient and (b) 5-100% MeCN/H_2O (0.1% TFA), 30 min gradient.

Table S2. Related to Figure 2. Assessment of models considered for the demethylation of H3 K4meK9[a)]

Model[a)]	CoREST	$InsP_6$ [b)]	MS275[c)]	Total normalised χ^2 [d)]	p-value [e)] w.r.t. Model (i)	p-value [e)] w.r.t. Model (ii)	p-value [e)] w.r.t. Model (iv)
	χ^2 / χ^2_{red}						
**(i)**	61.7/0.54	51.0/0.60	333/2.94	1251.4			
**(ii)**	49.7/0.44	49.9/0.59	190/1.69	812.9	2.1×10^{-32}		
**(iii)**	60.7/0.56	47.3/0.59	332/3.07	1234.7	0.501	N/A	
**(iv)**	32.7/0.29	48.3/0.58	123/1.11	572.8	7.1×10^{-56}	1.6×10^{-26}	
**(v)**	28.9/0.26	48.4/0.60	37.4/0.34	322.0	1.2×10^{-92}	1.8×10^{-63}	5.0×10^{-40}

a) The models considered are shown in Figure S4A. b) The CoREST complex was pre-equilibrated with 100 μM $InsP_6$. (c) The CoREST complex was pre-equilibrated with 5 μM MS275 HDAC inhibitor. (d) The sum of the χ^2 calculated for CoREST only, CoREST with $InsP_6$, and CoREST with MS275. Furthermore, the χ^2 was normalised as described by (Press et. al., 1992). (e) The probability value (significance), p-value, was calculated using F-test (Press et al., 1992). Model (v) is the most significant of the models considered.

Table S3. Related to Figure 2. Assessment of models considered for the deacetylation of H3 K4K9ac[a)]

	CoREST	$InsP_6$ [b)]	2-PCPA[c)]	Total normalised χ^2 [d)]	p-value [e)] w.r.t. model (i)	p-value [e)] w.r.t. model (ii)	p-value [e)] w.r.t. model (iv)
Model[a)]	χ^2 / χ^2_{red}						
**(i)**	1203/8.1	1675/12	1014/7.3	752.6			
**(ii)**	1167/7.9	1575/11	760/5.5	677.1	6.6×10^{-12}		
**(iii)**	1012/7.1	1666/12	963/7.2	704.1	1.8×10^{-5}	N/A	
**(iv)**	691/4.7	1391/9.9	519/3.8	502.9	1.1×10^{-39}	1.4×10^{-30}	
**(v)**	512/3.6	1317/9.6	462/3.5	443.0	9.5×10^{-50}	1.6×10^{-40}	6.3×10^{-13}

a) The models considered are shown in Figure S4A. b) The CoREST complex (50 nM) was pre-equilibrated with 100 μM $InsP_6$. c) The CoREST complex was pre-equilibrated with 5 μM MS275 HDAC inhibitor. d) The sum of the χ^2 calculated for CoREST only, CoREST with $InsP_6$, and CoREST with MS275. Furthermore, the χ^2 was normalised as described by by (Press et. al., 1992). e) The probability value (significance), p-value, was calculated using F-test by (Press et. al., 1992). Model (v) is the most significant of the models considered.

Table S4. Related to Table 1. Full list of kinetic parameters obtained for the demethylation of H3 K4meK9 substrate by CoREST.

	**Protein only**	**$InsP_6$[a)]**	**MS275[b)]**	**2-PCPA[c)]**
**χ^2 / χ_{red}^2**	28.8 / 0.26	48.2 / 0.60	37.6 / 0.35	43.7 / 0.43
**$K_{m,E}$ (μM)**	30 ± 21	2390 ± 230	550± 300	394 ± 81
**$k_{cat,E}$ (s^{-1})**	2.5 ± 1.0	4.61 ± 0.15	3.4 ± 0.9	0.97 ± 0.05
**$k_{cat,E} / K_{m,E}$($s^{-1}\mu M^{-1}$)**	0.083 ± 0.047	0.0019 ± 0.0002	0.0070 ± 0.0022	0.0025 ± 0.0002
**$K_{m,E*}$ (μM)**	1.6 ± 1.4	146 ± 25	5.4 ± 1.1	23 ± 5
**$K_{m,post}$ (μM)**	2.9 ± 1.7	193 ± 30	9.3 ± 1.6	25.7 ± 5.5
**$k_{cat,post}$ (s^{-1})**	0.032 ± 0.002	0.093 ± 0.007	0.023 ± 0.003	0.0077 ± 0.0012
**K_{eq}(E)**	7.0 ± 2.4	3.0 ± 0.3	1.7 ± 0.6	7.4 ± 0.5
**k_{ex}(E) (s^{-1})**	0.0074 ± 0.0024	0.0015 ± 0.0013	0.0028 ± 0.0015	$< 5 \times 10^{-4}$
**K_{eq}(ES)**	76 ± 28	49 ± 4	146 ± 38	129 ± 30
**k_{ex}(ES) (s^{-1})**	0.0055 ± 0.0012	0.047 ± 0.020	0.0060 ± 0.0024	0.0050 ± 0.0008

(a) The CoREST complex was pre-equilibrated with 100 μM $InsP_6$. (b) The CoREST complex was pre-equilibrated with 5 μM MS275 HDAC inhibitor. (c) The CoREST complex was pre-equilibrated with 100 μM 2-PCPA LSD1 inhibitor.

Table S5. Related to Table 2. Full list of kinetic parameters obtained for the deacetylation of H3K9ac substrate by CoREST.

	**Protein only**	**$InsP_6$[a]**	**2-PCPA[b]**
**χ^2 / χ_{red}^2**	513 / 3.6	1300 / 9.6	462 / 3.5
**$K_{m,E}$ (μM)**	10570 ± 5700	10640 ± 6700	1190 ± 960
**$k_{cat,E}$ (s^{-1})**	440 ± 290	510 ± 350	45 ± 33
**K_{m,E^*} (μM)**	24 ± 5	5.7 ± 4.2	16 ± 3
**k_{cat,E^*} (s^{-1})**	0.52 ± 0.03	0.4 ± 0.2	0.33 ± 0.06
**$K_{m,post}$ (μM)**	33 ± 6	12 ± 5	23 ± 4
**$k_{cat,post}$ (s^{-1})**	0.70 ± 0.03	0.78 ± 0.18	0.52 ± 0.05
**K_{eq}(E)**	6.8 ± 2.3	1.9 ± 2.1	5.5 ± 2.2
**k_{ex}(E) (s^{-1})**	$< 10^{-7}$	0.14 ± 0.17	0.0031 ± 0.0017
**K_{eq}(ES)**	2560 ± 1620	1990 ± 1600	280 ± 220
**k_{ex}(ES) (s^{-1})**	0.077 ± 0.042	0.094 ± 0.066	0.0028 ± 0.0016

(a) The CoREST complex was pre-equilibrated with 100 μM $InsP_6$. (b) The CoREST complex was pre-equilibrated with 100 μM 2-PCPA LSD1 inhibitor.

Table S6. Related to Figure 2. Combined analyses of K4meK9, K4K9ac and K4meK9ac using time points up to 2000 s.

Model no.	Comments	χ^2 / χ_{red}^2
1 [a]	*Completely uncoupled reactions:* It is assumed that the HDAC and LSD1 are completely uncoupled, that both demethylation and deacetylation reactions follow Michaelis-Menten and that (*a*) demethylation of K4meK9ac follows the same parameters as K4meK9 (*b*) deacetylation of K4meK9ac follows the same parameters as K4K9ac.	10706 / 20.5
2	*Semi-uncoupled reactions:* It is assumed that the HDAC and LSD1 functions independently, but that there is a substrate dependence. Thus, different Michaelis-Menten parameters are assumed for demethylation of K4meK9ac and K4meK9 and different Michaelis-Menten parameters are assumed for deacetylation of K4meK9ac and K4K9ac.	7147 / 13.8
3	*Semi-coupled reactions:* It is assumed that CoREST can only bind one substrate at once. Thus, if substrate is bound to LSD1 then substrate cannot bind to HDAC. Michaelis-Menten parameters are independent on substrate.	12991 / 24.9
4 [a]	*Coupled reactions:* It is assumed that CoREST can only bind one substrate at once. Substrate dependent Michaelis-Menten parameters are assumed.	2570 / 4.96
5	*Semi-uncoupled reactions with product inhibition.* Same as model 2 but with product inhibition, assuming different dissociation constants, K_i, for (*a*) LSD1 inhibition by K4K9ac (*b*) LSD1 inhibition by K4K9 (*c*) HDAC inhibition by K4meK9 and (*d*) HDAC inhibition by K4K9.	3111 / 6.05
6	*Semi-coupled reactions with product inhibition.* Same as Model 3 but with product inhibition as in Model 5.	3731 / 7.19
7	*Coupled reactions with product inhibition.* Same as Model 4, but with product inhibition.	2529 / 4.91
8 [a]	*Coupled reaction with alternate state of enzyme-substrate complexes (Figure S6).* Assuming $k_{\pm 3L} = k_{\pm 3La} = k_{\pm 3H} = k_{\pm 3Hm}$	1981 / 3.84
9 [a]	*Coupled reaction with alternate state of enzyme-substrate complexes (Figure S6).* Assuming all $k_{\pm 3L} = k_{\pm 3La}$, $k_{\pm 3H} = k_{\pm 3Hm}$	1614 / 3.14
10 [a]	*Coupled reaction with alternate state of enzyme-substrate complexes (Figure S6).*	1589 / 3.10
11 [a]	*Coupled reaction with alternate state of the enzyme-substrate complexes and product inhibition.*	1587 / 3.12
12	*Coupled reaction with alternate state of the free enzyme and enzyme-substrate complexes*	Not converged.

(a) The following *p*-levels are obtained from comparisons of the chi-squared, χ^2, and the degrees of freedoms of the fits. Model 1 *v.s.* Model 4: *p*-level $<10^{-50}$, Model 2 *v.s.* Model 4: Model 4 *v.s.* Model 8: *p*-level = 7×10^{-30}, Model 9 *v.s.* Model 8: *p*-level = 1×10^{-23}, Model 10 *v.s.* Model 9: *p*-level = 0.018, Model 11 *v.s.* Model 10: *p*-level = 0.89. The most significant model is Model 10, that is, a coupled reaction with different rate constants for the different substrates and an exchange of the enzymes with alternate states.

Table S7. Related to Figure 3. Data collection and structure statistics for small angle X-ray scattering analysis

**Sample properties**	
Organism	Homo sapiens
Solvent	25 mM Tris pH 7.5, 50 mM potassium acetate and 0.5 mM TCEP
Components	RCOR1 (86-485)<br>HDAC1<br>LSD1
**Data collection parameters**	
Instructment	Diamond light source B21 (Didcot, UK)
Beam geometry (mm^2)	0.8 × 2 mm
Wavelength (Å)	~ 1
s range ($Å^{-1}$)	0.0032 - 0.38
Exposure time (s)	5
Temperature (K)	298
**Structural parameters**	
Rg (Å) (from P(r))	58.14
Rg (Å) (from Guinier plot)	60
I(0) (from P(r))	0.00682
I(0) (from Guinier plot)	0.00825
Dmax (Å)	158
Porod volume estimate ($Å^3$)	437000
**Molecular mass determination (kDa)**	
From Porod volume	257
From SAXS MoW	265
Calculated molecular mass from sequence	194
**Software employed**	
Primary data reduction	ScÅtter
Data processing	ScÅtter
Ab initio analysis	ScÅtter/DAMIF
Validation and averaging	ScÅtter/DAMAVER
Atomic structure modelling	CORAL
Computation of model intensities and fitting with experimental data	CRYSOL
3D graphics representations	Pymol
