# Supplementary material for: Mechanism of Crosstalk between the LSD1 Demethylase and HDAC1 Deacetylase in the CoREST Complex
Source: Cell Rep. 2020 Feb 25;30(8):2699–2711.e8. doi: 10.1016/j.celrep.2020.01.091 (PMC7043024; doi:10.1016/j.celrep.2020.01.091)
Supplement: Document S2. Article plus Supplemental Information [file mmc2.pdf]

Cell Reports

Article

Mechanism of Crosstalk between the LSD1 Demethylase and HDAC1 Deacetylase in the CoREST Complex

Graphical Abstract

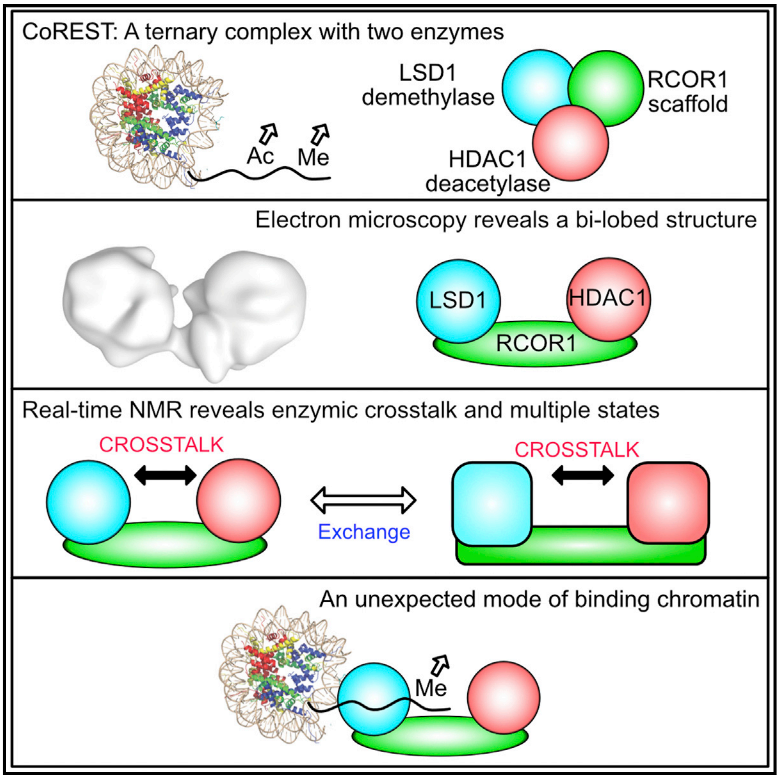

Highlights

- The activities of LSD1 and HDAC1 are closely coupled in the CoREST complex
- Both LSD1 and HDAC1 exist in two different kinetic states
- CoREST has a bi-lobed, flexible structure with the two enzymes located at opposite ends
- CoREST interacts with methylated nucleosomes via LSD1, but not HDAC1 or RCOR1

Authors

Yun Song, Lisbeth Dagil, Louise Fairall, ..., Philip A. Cole, D. Flemming Hansen, John W.R. Schwabe

Correspondence

d.hansen@ucl.ac.uk (D.F.H.),
john.schwabe@le.ac.uk (J.W.R.S.)

In Brief

Using a real-time NMR assay, Song et al. characterize crosstalk between LSD1 and HDAC1 in the CoREST complex. Activation or inhibition of one enzyme strongly affects activity of the other. Electron microscopy studies of the complex reveal a bi-lobed structure with implications for the mode of interaction with nucleosomes.

Song et al., 2020, Cell Reports *30*, 2699–2711
February 25, 2020 © 2020 The Author(s).
https://doi.org/10.1016/j.celrep.2020.01.091

Cell Reports
Article

OPEN ACCESS
CellPress

Mechanism of Crosstalk between the LSD1 Demethylase and HDAC1 Deacetylase in the CoREST Complex

Yun Song,[1,6] Lisbeth Dagil,[2,7] Louise Fairall,[1] Naomi Robertson,[3,8] Mingxuan Wu,[4,10] T.J. Ragan,[1] Christos G. Savva,[1] Almutasem Saleh,[1,11] Nobuhiro Morone,[5] Micha B.A. Kunze,[2,7] Andrew G. Jamieson,[3,9] Philip A. Cole,[4] D. Flemming Hansen,[2,*] and John W.R. Schwabe[1,12,*]
[1]Leicester Institute of Chemical and Molecular Biology, Department of Molecular and Cell Biology, University of Leicester, Lancaster Road, Leicester LE1 7RH, UK
[2]Institute of Structural and Molecular Biology, Division of Biosciences, University College London, Gower Street, London WC1E 6BT, UK
[3]Department of Chemistry, University of Leicester, University Road, Leicester LE1 7RH, UK
[4]Division of Genetics, Department of Medicine, Brigham and Women's Hospital and Department of Biological Chemistry and Molecular Pharmacology, Harvard Medical School, Boston, MA 02115, USA
[5]MRC-Toxicology Unit, University of Cambridge, University Road, Leicester LE1 7RH, UK
[6]Present address: Electron Bio-Imaging Centre, Diamond Light Source, Didcot OX11 0DE, UK
[7]Present address: Novo Nordisk A/S, Novo Nordisk Park, 2760 Måløv, Denmark
[8]Present address: Department of Biochemistry and Department of Chemistry, University of Cambridge, Cambridge CB2 1GA, UK
[9]Present address: School of Chemistry, Joseph Black Building, University of Glasgow, University Avenue, Glasgow G12 8QQ, UK
[10]Present address: Westlake University, Hangzhou, Zhejiang, China
[11]Present address: Institute of Clinical Sciences, Faculty of Medicine, Imperial College London, London W12 0HS, UK
[12]Lead Contact
*Correspondence: d.hansen@ucl.ac.uk (D.F.H.), john.schwabe@le.ac.uk (J.W.R.S.)
https://doi.org/10.1016/j.celrep.2020.01.091

SUMMARY

The transcriptional corepressor complex CoREST is one of seven histone deacetylase complexes that regulate the genome through controlling chromatin acetylation. The CoREST complex is unique in containing both histone demethylase and deacetylase enzymes, LSD1 and HDAC1, held together by the RCOR1 scaffold protein. To date, it has been assumed that the enzymes function independently within the complex. Now, we report the assembly of the ternary complex. Using both structural and functional studies, we show that the activity of the two enzymes is closely coupled and that the complex can exist in at least two distinct states with different kinetics. Electron microscopy of the complex reveals a bi-lobed structure with LSD1 and HDAC1 enzymes at opposite ends of the complex. The structure of CoREST in complex with a nucleosome reveals a mode of chromatin engagement that contrasts with previous models.

INTRODUCTION

The molecular machinery that installs and removes post-translational modifications of chromatin has been the subject of increasing research interest not least because these protein complexes are key regulators of gene expression, but they are also promising drug targets for the epigenetic treatment of cancer and other diseases (Delcuve et al., 2012; Hesham et al., 2018; Millard et al., 2017; Rowe et al., 2019). It is now well established that acetylation and methylation of lysine residues within the tails of histone proteins not only control the recruitment of regulatory factors but also influence the architecture of chromatin itself. The CoREST complex is one of seven families of class I histone deacetylase complexes that have specialized physiological functions but are all thought to act as repressors of gene expression. CoREST is unique within these complexes in that it removes both acetyl and methyl modifications through the activity of its demethylase (LSD1) and deacetylase (HDAC1) enzymes.

The CoREST complex was initially identified as a cofactor of the REST/NRSF (RE1-silencing transcription factor/neural-restrictive silencer factor) transcriptional repressor, which plays important roles in regulating neuron-specific gene expression and stem cell fate and development (Andrés et al., 1999; Ballas et al., 2001; Foster et al., 2010; Wang et al., 2007). The core of the CoREST complex contains two histone modification enzymes, including histone deacetylase 1 or 2 (Humphrey et al., 2001; You et al., 2001), lysine specific demethylase LSD1 (Lee et al., 2005; Shi et al., 2004, 2005), and RCOR1, 2, or 3 that links the two enzymes. The classic enzymatic target for the CoREST complex is the histone H3 tail in which K4 is mono- or di-methylated and K9 is acetylated. Removal of these activating marks results in transcriptional repression (Andrés et al., 1999; Lakowski et al., 2006; Lee et al., 2005; You et al., 2001).

The RCOR protein serves as a scaffold for complex assembly and the recruitment of the complex to the repressive transcription factors (Humphrey et al., 2001; Saleque et al., 2007; Shi et al., 2005; You et al., 2001). RCOR contains an ELM2-SANT1 domain that mediates interaction with the catalytic domain of HDAC1 (You et al., 2001) and a LINKER-SANT2 domain that

This is an open access article under the CC BY license (http://creativecommons.org/licenses/by/4.0/).

interacts with the TOWER domain of LSD1 (Shi et al., 2005; Figure 1A). The RCOR-SANT2 domain has also been proposed to directly interact with nucleosomal DNA (Pilotto et al., 2015; Yang et al., 2006).

Multiple crystal structures of LSD1 in complex with the LINKER-SANT2 of RCOR have revealed the mode of assembly and the nature of the LSD1 active site and its interaction with substrates and inhibitors (Baron et al., 2011; Forneris et al., 2007; Yang et al., 2006, 2007). There is no structure of HDAC1 in complex with RCOR, but a homologous structure of HDAC1 bound to the ELM2-SANT domain of MTA1 likely has many features in common (Millard et al., 2013).

Given the dual functionality of the CoREST complex, we sought to investigate the relationship between the two different enzymes when assembled together. Such an integrated understanding of the ternary complex has, to date, been lacking. We have taken a structural approach to understand the relative positioning of the two enzymes in the complex and an enzymatic approach to explore potential crosstalk between the two activities. We have found that, although the two enzymes are positioned at either ends of the complex, there is a remarkable coupling between their enzymatic activities. Inhibitors of one enzyme strongly influence the kinetics of the partner enzyme, and only one active site can engage substrate at any one time. It is also apparent that the complex exists in at least two distinct states. Exchange between these is sensitive to modulators of the complex. Finally, the structure of CoREST in complex with a nucleosome, in which histone H3K4 is modified with a propargyl inhibitor, reveals a mode of binding that is distinct from previous models.

RESULTS

RCOR1, LSD1, and HDAC1 Form a Stable, Enzymatically Active, Stoichiometric Ternary Complex

Studies of the CoREST complex to date have investigated the independent activities of the demethylase and deacetylase enzymes. To understand the behavior of these enzymes in the context of the full ternary CoREST complex, we have co-expressed full-length LSD1 and HDAC1 and a construct of the RCOR1 corepressor (amino acids [aas] 86–485) lacking only the N-terminal disordered region. The holo CoREST ternary complex was purified to homogeneity using an N-terminal FLAG affinity tag followed by size exclusion chromatography (Figure 1B).

In our previous studies, we have observed that corepressor complexes containing class I HDACs (SMRT/NCoR, NuRD, and MiDAC) assemble into either dimeric or tetrameric complexes (Itoh et al., 2015; Millard et al., 2013; Oberoi et al., 2011). Detailed structural information is available for the crystal structure of the dimeric MTA1:HDAC1 complex (Millard et al., 2013)—see alignment (Figure 1C). To determine the oligomerization state of the CoREST complex, we used size exclusion chromatography coupled with multi-angle light scattering (SEC-MALS). The overall molecular weight was measured to be ~200 kDa (Figure 1D), which correlates with the predicted monomeric molecular weight of 193 kDa (full-length LSD1, 92.9 kDa; full-length HDAC1, 55.1 kDa; RCOR1$_{86–485}$, 45.1 kDa), indicating that the full ternary CoREST complex contains a single copy of HDAC1, LSD1, and RCOR1. A sequence comparison of RCOR1–3 with MTA1–3 shows that the ELM2 domain within RCOR1 lacks the helices that mediate dimerization of MTA1 in the NuRD corepressor complex (Figure 1C).

To confirm that the ternary complex that we expressed and purified from HEK293 cells is active, we used fluorogenic enzyme assays to measure both the deacetylase and demethylase activity of the complex (Figures 1E and 1F). As controls, we used the HDAC inhibitors SAHA and MS275 and the demethylase inhibitors 2-PCPA and SP2509 to confirm that the observed activity could be inhibited as expected. Several class I HDAC complexes are activated by inositol phosphates that bind in a pocket between the corepressor and the HDAC, close to the active site (Itoh et al., 2015; Millard et al., 2013; Watson et al., 2012, 2016). Inspection of the sequence of RCOR1 suggests that the inositol phosphate binding residues are conserved, and therefore, we would predict that the CoREST complex would also interact with inositol phosphates (Figure S1A). To test this, we measured the deacetylase activity of the ternary complexes in the absence and presence of Ins(1,4,5,6)P_4 and InsP_6. We observed a significant increase in HDAC activity in the presence of both inositol phosphates, suggesting that these may regulate the HDAC activity of the CoREST complex (Figure 1E). As for other class I HDAC complexes, Ins(1,3,4,5,6)P_5 also activates HDAC1 in the CoREST complex (Figure S1B; Watson et al., 2016).

Coupled Kinetics of the LSD1 and HDAC1 Enzymes within the CoREST Complex

To determine whether the LSD1 and HDAC1 enzymes within the CoREST complex behave as independent enzymes or whether they are coupled, a ^{1}H NMR-based assay was developed. In this assay, demethylation and deacetylation of 21-amino-acid synthetic peptides corresponding to the N terminus of histone H3 (with specific post-translational modifications; Table S1) are monitored in real time. Initially, we used singly modified peptides containing either mono-methyl K4 (K4meK9) or acetyl K9 (K4K9ac). Peaks corresponding to the methyl protons of K4me, K9ac, and the free acetate product could readily be observed and distinguished in one-dimensional ^{1}H NMR spectra (Figures 2A, 2B, 2F, and 2G). The HDAC and LSD1 inhibitors and inositol phosphates used in these experiments do not contain peaks that overlap with the methyl or acetyl peaks. Both demethylase and deacetylase reactions were monitored, with a range of initial H3 substrate concentrations, by using the intensities of the K4me and K9ac peaks as reporters on the concentration of the substrates in real time.

Analysis of the progression curves (Figures 2C, 2D, 2H, 2I, S2, and S3) for the demethylation of the K4meK9 substrate and the deacetylation of K4K9ac showed that the reactions do not follow classical Michaelis-Menten kinetics, but the apparent K_M and k_{cat} parameters are strongly time dependent with biphasic progression curves. This biphasic behavior is particularly pronounced for the demethylation reaction upon addition of the HDAC inhibitor MS275 and for the deacetylation reaction upon addition of the LSD1 inhibitor 2-PCPA. A number of different reactions schemes were considered, including those involving standard substrate and product inhibition (Figure S4A; Tables S2 and S3). The simplest reaction scheme, which overall gave satisfactory results for both the demethylation and deacetylation

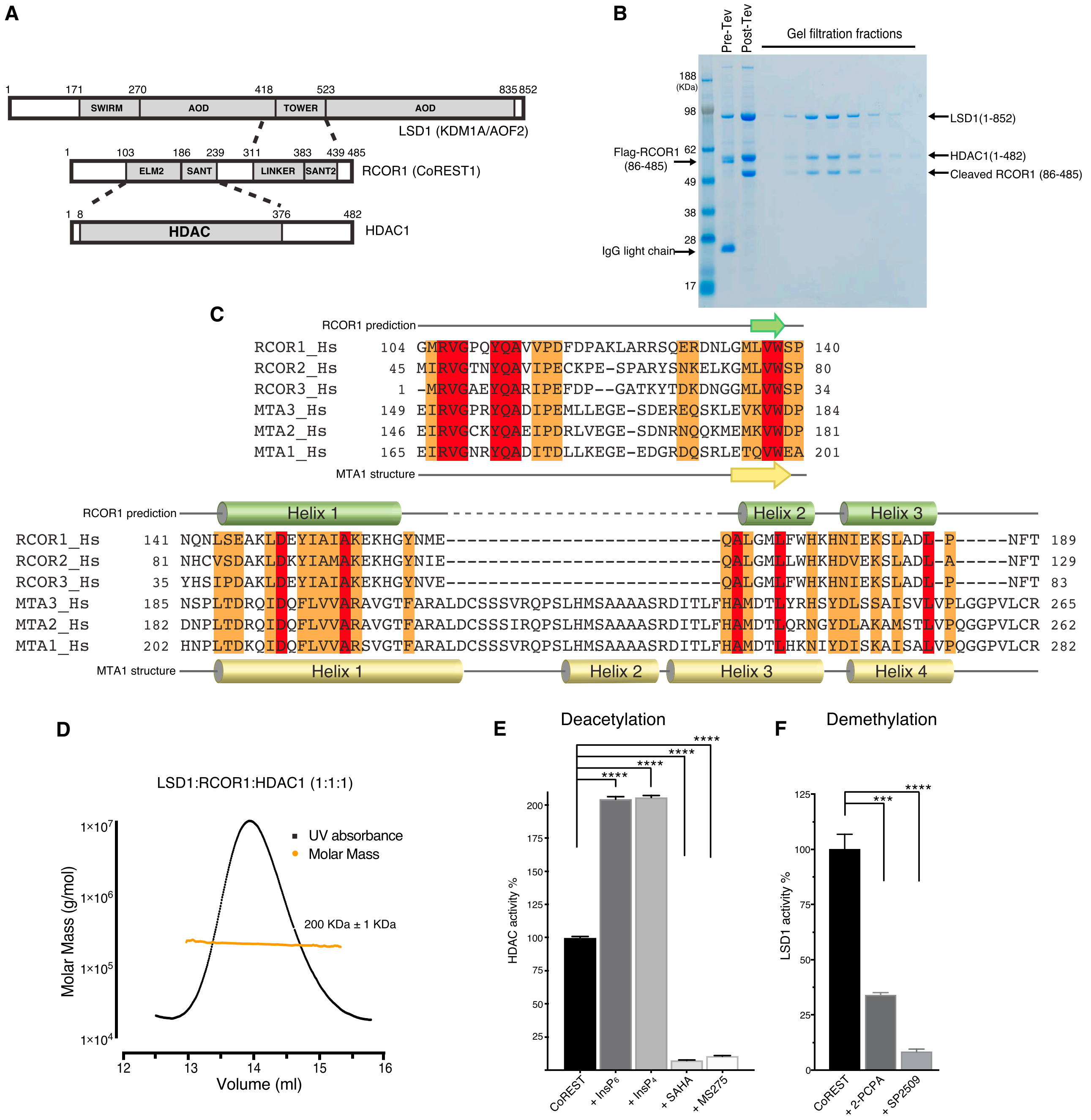

Figure 1. The CoREST Complex Forms a Stable, Enzymatically Active, and Stoichiometric Complex

(A) Schematic representation of domain structures of LSD1/KDM1A/AOF2, CoREST/RCOR1, and HDAC1. Gray boxes represent the structured domains. Dashed lines indicate the interacting regions within the complex.

(B) Co-expression and purification of the LSD1:RCOR1:HDAC1 ternary complex.

(C) Sequence alignment of the ELM2 domain from RCOR1–3 and MTA1–3 proteins. Identical residues are shown in red, and conserved residues are shown in orange. The predicted secondary structure of RCOR1 is indicated above the sequence (green), and the secondary structure of MTA1 observed in the crystal structure is indicated below the sequence (yellow).

(D) Stoichiometry/molecular weight determination of the CoREST ternary complex by SEC-MALS.

(E) Deacetylase activity of the ternary complex. As expected, the activity is enhanced by 100 μM Ins(1,4,5,6)P_4 ($InsP_4$) and by Ins(1,2, 3,4,5,6)P_6 ($InsP_6$). The activity is inhibited by SAHA and MS275 (5 μM). The activity is normalized (100%) to the basal HDAC activity. The basal activity of the assay with no complex has been subtracted. Error bars indicate the SEM (n = 3). p values are shown in the form: *** $p < 0.001$ or **** $p < 0.0001$.

(F) Demethylase activity of the ternary complex. As expected, the activity is inhibited by 2-PCPA and SP2509 (10 μM). The activity is normalized (100%) to the basal demethylase activity. The basal activity of the assay with no complex has been subtracted. Error bars indicate the SEM (n = 3). p values are shown in the form: *** $p < 0.001$ or **** $p < 0.0001$.

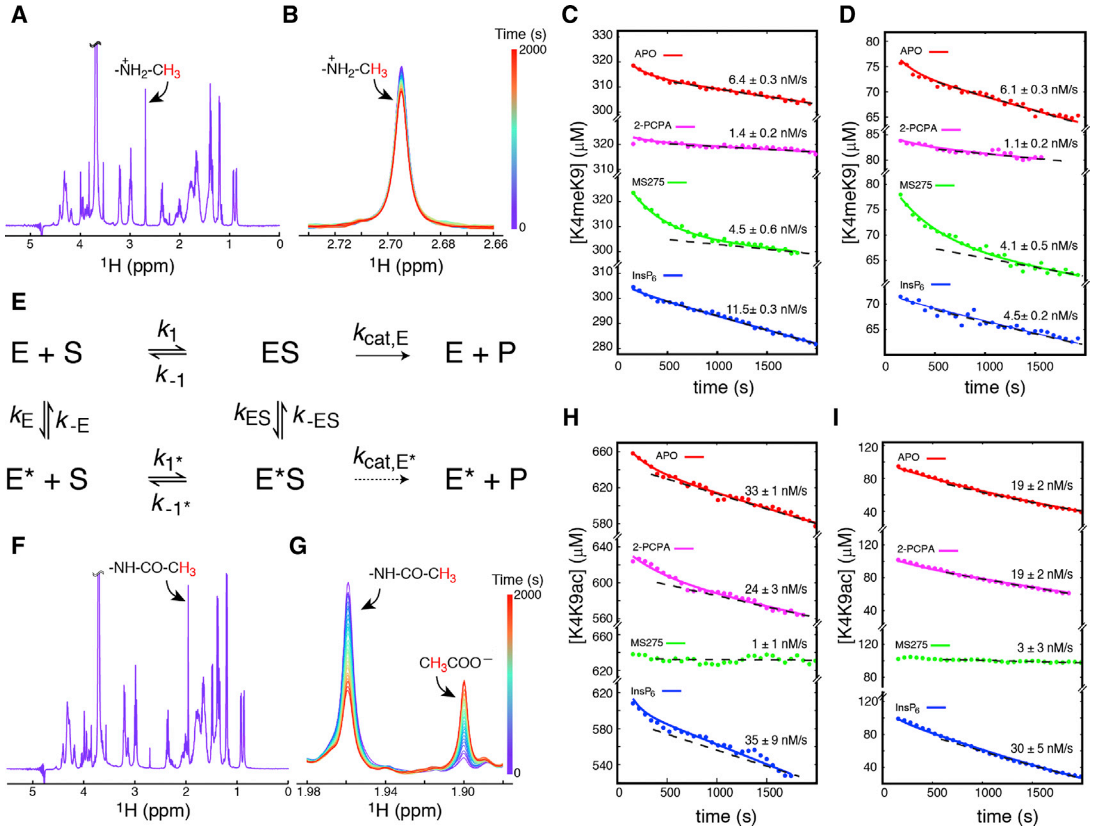

Figure 2. Enzymatic Coupling between LSD1 and HDAC1 in the CoREST Complex

(A) The 1H NMR reference spectrum of a 330 μM sample of H3K4me, with the assignment of the K4 N(6) methyl protons shown.

(B) Time series after the addition of 200 nM CoREST complex to the H3K4me substrate.

(C and D) Progression curves for the conversion of ca. 300 μM (C) or 80 μM (D) H3K4me substrate incubated with 200 nM CoREST complex.

(E) Filled circles are experimentally obtained substrate concentrations versus time, full-drawn lines are the results of the least-squares fits to the reaction scheme, and dashed lines represent the limiting rates after equilibrium is reached.

(F) Reference 1H NMR spectrum of 670 μM H3K9ac with the assignment of the methyl protons of the K9 acetyl group shown.

(G) A representative time series obtained after the addition of 50 nM CoREST complex to a 100 μM sample of H3K9ac. It is noted that both the disappearance of the H3K9ac substrate and the appearance of the acetate product can be observed, and their concentrations can be quantified from the intensity of the two peaks.

(H and I) Progression curves for ca. 660 μM (H) or 100 μM (I) H3K9ac substrate concentration versus time after addition of 50 nM CoREST.

reactions, is shown in Figure 2E. The key feature of this reaction scheme is that it invokes an alternate state (E*) of the enzymes (HDAC1 and LSD1), which exhibit different enzymatic parameters. In particular, the first-order catalytic rate, k_{cat}, and the substrate disassociation rate, k_{-1}, are different for E and E*. Moreover, the model suggests a slow unimolecular exchange between E and E* and between ES and E*S. For all demethylation reactions, the catalytic rate from E*S was insignificant ($p \sim 0.8$; $k_{cat,E}{}^{*} \sim 0\ s^{-1}$), although $k_{cat,E}{}^{*} > 0\ s^{-1}$ was significant for all the deacetylation reactions (p of 0.003–0.05). The existence of an alternate state of the HDAC1 and LSD1 enzymes means that the overall reaction is highly dependent upon whether equilibrium has been reached between ES and E*S. When equilibrium between ES and E*S has been reached, apparent Michaelis-Menten parameters can be calculated (Supplemental Information), which are denoted $K_{M,post}$ and $k_{cat,post}$ (Tables 1, 2, S4, and S5). The dashed lines in Figures 2C, 2D, 2H, and 2I are calculated based on the limiting condition of long reaction times and ES $\rightleftharpoons$ E*S equilibrium.

As might be expected, addition of the 2-PCPA LSD1 inhibitor increases the $K_{M,post}$ of the demethylation reaction by approximately an order of magnitude and also decreases the catalytic rate by a factor of three, in agreement with the results of the fluorogenic assay in Figure 1F. Of particular interest is that the addition of the HDAC inhibitor MS275 has a profound effect on both the progression curves (Figures 2C and 2D) and kinetic parameters, obtained for the demethylation of K4meK9. The catalytic rate post-equilibrium, $k_{cat,post}$, is only marginally affected, as is the equilibrium between ES and E*S. However, $K_{M,post}$ is about a factor of four larger and the rate E → E* is a factor of three slower. This change clearly indicates a coupling between LSD1 and HDAC1 within the CoREST complex and leads to a slower convergence toward the $K_{M,post}$ and $k_{cat,post}$ parameters, which is clearly visible in the experimental progression curves. These findings indicate a negative regulation, where once inhibitor or substrate is bound to HDAC1, the binding affinity for substrate to LSD1 decreases, which is also in agreement with previous observations that trichostatin A inhibited demethylation of

Table 1. Summary of Kinetic Parameters Obtained for the Demethylation of K4meK9 by CoREST

	APO	2-PCPA[a]	MS275[b]	$InsP_6$[c]
Post-equilibrium Apparent Parameters				
$k_{cat,post}$ (s^{-1})	0.032 ± 0.002	0.0077 ± 0.0012	0.023 ± 0.003	0.093 ± 0.007
$K_{M,post}$ (μM)	2.3 ± 1.7	26 ± 5	9.2 ± 1.6	190 ± 30
$k_{cat,post}/K_{M,post}$ ($s^{-1}mM^{-1}$)	14 ± 7	0.30 ± 0.02	2.5 ± 0.4	0.48 ± 0.04
Unimolecular Exchange				
K_{eq}(E)	7.0 ± 2.4	7.4 ± 0.5	1.7 ± 0.6	3.0 ± 0.3
k_{ex}(E) (s^{-1})	0.0074 ± 0.0024	<5 × 10^{-4}	0.0028 ± 0.0015	0.0015 ± 0.0013
K_{eq}(ES)	76 ± 28	129 ± 30	146 ± 38	49 ± 4
k_{ex}(ES) (s^{-1})	0.0055 ± 0.0012	0.0050 ± 0.0008	0.0060 ± 0.0024	0.047 ± 0.020

[a]The 200 nM CoREST complex was pre-equilibrated with 0.1 mM 2-PCPA LSD1 inhibitor.
[b]The CoREST complex was pre-equilibrated with 5 μM MS275 HDAC inhibitor.
[c]The CoREST complex was pre-equilibrated with 0.1 mM $InsP_6$.

Table 2. Kinetic Parameters Obtained for the Deacetylation of K4K9ac by CoREST

	APO	2-PCPA[a]	$InsP_6$[b]
Post-equilibrium Apparent Parameters			
$k_{cat,post}$ (s^{-1})	0.70 ± 0.03	0.52 ± 0.05	0.78 ± 0.18
$K_{M,post}$ (μM)	33 ± 6	22 ± 4	12 ± 5
$k_{cat,post}/K_{M,post}$ ($s^{-1}mM^{-1}$)	21 ± 2	23 ± 3	70 ± 50
Unimolecular Exchange			
K_{eq}(E)	6.8 ± 2.3	5.5 ± 2.2	1.9 ± 2.1
k_{ex}(E) (s^{-1})	<10^{-7}	0.0031 ± 0.0017	0.14 ± 0.17
K_{eq}(ES)	2,560 ± 1,620	280 ± 220	1,990 ± 1,600
k_{ex}(ES) (s^{-1})	0.077 ± 0.042	0.0028 ± 0.0016	0.09 ± 0.07

[a]The CoREST complex was pre-equilibrated with 0.1 mM 2-PCPA LSD1 inhibitor.
[b]The CoREST complex was pre-equilibrated with 0.1 mM $InsP_6$.

nucleosomes (Lee et al., 2006). Addition of $InsP_6$ also has a substantial effect on the demethylase progression curves and derived kinetic parameters. Most remarkable is an increase of about two orders of magnitude in the post-equilibrium apparent Michaelis-Menten constant for $K_{M,post}$ and an order of magnitude increase in the k_{ex}(ES) rate such that the equilibrium between ES and E*S is reached nearly within the dead time of the experiment. The obtained parameters means that, at high concentrations of substrate ($[S]_0$ > 200 μM), addition of $InsP_6$ increases the effective rate, although for lower substrate concentrations ($[S]_0$ < 100 μM), addition of $InsP_6$ slows the effective reaction rate.

The progression curves for the deacetylation of K4K9ac by CoREST are shown in Figures 2H and 2I for apo CoREST and for CoREST pre-incubated with the three modulators 2-PCPA, MS275, and $InsP_6$. The progression curves were analyzed using the same reaction scheme as shown in Figure 2E, with the exception of the experiments in which CoREST is incubated with the HDAC inhibitor MS275, for which no reliable deacetylase parameters could be obtained. The equilibrium constants between E and E* are essentially identical for the demethylation and the deacetylation reactions under the various conditions (Tables 1 and 2). The addition of $InsP_6$ results in an increase of $k_{cat,post}/K_{M,post}$ by a factor of three, which is in agreement with the results from the fluorogenic assay in Figure 1E. Most substantial is that the addition of $InsP_6$ leads to a dramatic increase in the exchange rate between E and E*. This effect is in line with the demethylation reaction where the addition of $InsP_6$ leads to a substantial change in the exchange rates between both ES ⇌ E*S and E ⇌ E*.

Although both the deacetylation and the demethylation reactions are best described by the reaction scheme in Figure 2E, the alternate states, E and E*, need not necessarily be the same for the two enzymes. The substantial downregulation by an HDAC inhibitor of the demethylation reaction clearly points to the fact that the two reactions/enzymes are coupled within the CoREST complex, and it seems likely that there is some form of structural coupling between the enzymes.

The coupling between the demethylation and deacetylation reactions was further substantiated by using a doubly modified substrate, K4meK9ac (Figure S4B). In this assay, the demethylation reaction is monitored at the same time as the deacetylation reaction in real time using the NMR-based assay. The progression curves for K4meK9, K4K9ac, and K4meK9ac were analyzed simultaneously with successively more complicated reaction schemes (Table S6). The analyses and subsequent F tests show that, with a very high confidence level, the two reactions are coupled. Specifically, (1) the CoREST complex binds only one substrate at once ($p < 10^{-100}$; model 2 versus model 4; Table S6; Supplemental Information). Thus, the HDAC1 enzyme cannot bind substrate when substrate is already bound to the LSD1 enzyme and vice versa. (2) Both the demethylation and deacetylation reactions depend on the specific substrate ($p < 10^{-100}$; model 3 versus model 4). That is, the kinetic parameters for deacetylation of K4meK9ac are different from those of K4K9ac and the kinetic parameters for demethylation of K4meK9ac are different from those of K4meK9—consistent with previous studies with isolated LSD1, which suggest that acetylation of K9 inhibits demethylation of K4 (Forneris et al., 2005). (3) The CoREST complex exchanges between alternate states ($p < 10^{-30}$; model 4 versus models 8–10). The reaction scheme with the most significant parameters, where proper convergence could be obtained, is shown in Figure S4C.

Overall, the kinetic analyses of the two enzymes within the CoREST complex reveal that they do not act independently and that their activity and modulation by inhibitors and activators is closely coupled.

Structural Relationship between LSD1 and HDAC1 within the CoREST Complex

Given that there appears to be coupling between the enzymatic activities of LSD1 and HDAC1 in the CoREST complex, we sought to gain an understanding of the structural relationship of the two enzymes in the complex. Attempts to obtain crystals of the ternary complex were unsuccessful, suggesting that there may be some flexibility within the complex that inhibits crystal formation. In the absence of diffraction quality crystals, as a first step, we used small angle X-ray scattering to analyze the overall shape (“envelope”) of the CoREST complex. The small angle X-ray scattering (SAXS) envelope of the CoREST complex has an asymmetric bi-lobed architecture (Figures 3A and 3B). The crystal structures of HDAC1:MTA1 (Millard et al., 2013; PDB: 4BKX) and LSD1:RCOR1 (Forneris et al., 2007; PDB: 2V1D) could be readily positioned within the SAXS envelope with the SWIRM and AOD (amine oxidase domain) domains of LSD1 in one lobe and LSD1(TOWER), RCOR1(LINKER-SANT2), and HDAC1: MTA1(SANT1) in the other lobe. We used CORAL to refine the position of the crystal structures and to model terminal regions of the proteins as well as the RCOR1 linker so as to optimize the fit with the SAXS data (Petoukhov et al., 2012). The theoretical scattering curve calculated from this refined model of holo CoREST complex gave a reasonable agreement with the experimental data (Figure 3C). However, a Kratky plot of the SAXS data suggests that the complex may have conformational flexibility, limiting the quality of the fit (Figure 3D).

To test this model of the CoREST complex, we used cross-linking analysis together with mass spectrometry to identify interacting surfaces. The purified CoREST complex was cross-linked by an isotopically coded NHS-activated ester cross-linking reagent (Figure 3E). Liquid chromatography-mass spectrometry (LC-MS) analysis identified cross-linked residues (Figure 3F). Rather few cross-links that report on the relationship between HDAC1 and LSD1 were identified. However, a cross-link between lysine 220 in HDAC1 with lysine 447 in the LSD1 TOWER domain was particularly informative. This cross-link is clearly compatible with the SAXS-derived model of the CoREST ternary complex (Figure 3G).

Negative-stain electron microscopy was used to further investigate the architecture of the holo CoREST complex. Samples of a lightly cross-linked CoREST complex were applied to a carbon-coated copper grid and stained with uranyl acetate (see STAR Methods for details of sample preparation). The resulting transmission electron microscopy (TEM) images revealed a homogeneous sample that enabled straightforward manual particle picking in the EMAN2 software (Figure 4A). Two-dimensional class averages generated using Relion revealed a clear two-lobed complex joined by a linker (Figure 4B). This bi-lobed shape fits well with the SAXS envelope. A 3D envelope for the structure was generated using CryoSparc at an approximate resolution of 18 Å (Figures 4C and 4D). The coordinates for the known structure of the LSD1:RCOR1 complex and a model of the HDAC1:RCOR1 complex (based on the HDAC1:MTA1 structure) could readily be fitted into this envelope. In the resulting model, the HDAC1 is located at the far end of the TOWER domain adjacent to the SANT2 domain of RCOR1. In addition to the clearly defined bridge between the two lobes, formed from the TOWER domain of LSD1 and the LINKER domain of RCOR1, there is evidence of a second less substantial linker. This second linker can be rationalized given that there must be a connection between the C terminus of the RCOR-SANT1 domain and the N terminus of the coiled coil LINKER domain of RCOR1 (Figure 4E). However, due to the limited resolution, it is difficult to confidently orient HDAC1 and its bound SANT1 domain within the lower lobe of the structure. It is also likely that the SANT2 domain from RCOR1 may be repositioned somewhat, compared with the crystal structure, to accommodate the HDAC1/RCOR1-SANT complex.

Given the homogeneity of the complex on negative-stain electron microscopy (EM) grids, we were hopeful to determine a higher resolution structure using samples flash frozen in vitrified ice (cryoelectron microscopy [cryo-EM]). A variety of different EM grids were prepared using a range of different buffer and cross-linking conditions. Cryo-EM data of the CoREST complex, lightly cross-linked with glutaraldehyde, revealed once again a clear bi-lobed structure in the 2D class averages (Figure S5C). Calculation of a 3D model did not yield a structure with significantly increased resolution but revealed a clearly asymmetric bi-lobed envelope (Figure 4F). This allowed unambiguous fitting of the LSD1:RCOR1 crystal structure. The remaining density could accommodate the HDAC1:SANT in a similar position to the model fitted in the envelope obtained from the negative stain data.

We also collected cryo-EM data for the CoREST complex cross-linked with BS^3 using a Volta phase plate. The 2D class averages from these micrographs were rather less homogeneous (Figure S5D). Calculation of 3D models revealed two distinct conformations for the CoREST complex (Figure 4G). The first closely resembles the structure seen in both the negatively stained dataset and the glutaraldehyde cross-linked dataset in ice. In the second conformation, the HDAC1 is pivoted away from the coiled coil of the LSD1 TOWER. We refer to these as “closed” and “open” conformations. Interestingly, in the BS^3 cross-linked complex, in both frozen and negatively stained complex, we observed some class averages in which the HDAC1 was apparently fully detached from the LSD1 TOWER and relocated toward the LSD1(AOD) domain (Figure S5E). We believe that this is likely to be an artifact arising from partial disassembly of the complex as a result of the harsh grid preparation process. Furthermore, in the absence of cross-linking, it was only possible to observe isolated LSD1 on cryo-EM grids (Figure S5E), emphasizing that the freezing process is damaging to the integrity of the complex.

Structure of the CoREST Complex Bound to a Mononucleosome

Although we have been unable to determine a high-resolution structure of the CoREST complex, we sought to gain insights into the interaction of the holo-complex with a mono-nucleosome substrate. We took the strategy of preparing site-specific modified nucleosomes that contain a propargylamine mimic of histone H3 K4me2 (Culhane et al., 2006; Forneris et al., 2007). The nucleosome was assembled with 185-bp 601 DNA (Lowary and Widom, 1998), because it has been suggested that linker DNA is required for tight binding between CoREST and nucleosomes (Kim et al., 2015; Wu et al., 2018). This 185-bp H3K4-propargyl nucleosome

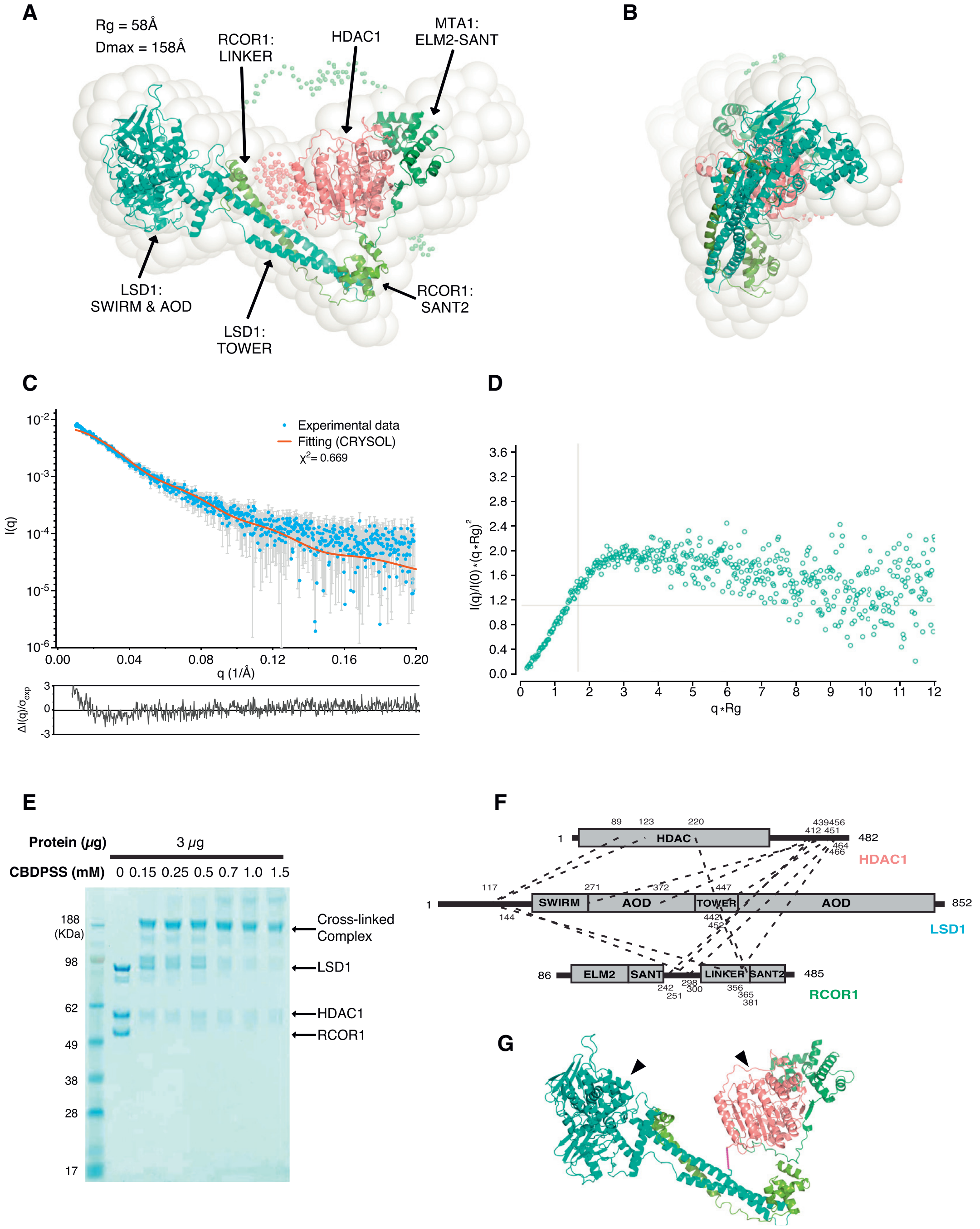

Figure 3. The CoREST Ternary Complex has a "Bi-lobe" Structure

(A) View of the SAXS envelope of the CoREST ternary complex fitted using CORAL with the crystal structures of the MTA1:HDAC1 (PDB: 4BKX; Millard et al., 2013) and LSD1:RCOR1 (PDB: 2V1D; Forneris et al., 2007) complexes. The linker and terminal disordered regions are modeled and indicated by small C-alpha spheres.

(B) View as in (A) but rotated by 90°.

(C) SAXS data for the CoREST ternary complex with the experimental scattering curve (blue) and theoretical scattering curve (red) from the LSD1:RCOR1:HDAC1 model. The residual errors of the fit are shown below the curve.

(D) A Kratky plot indicating that the CoREST complex is conformationally flexible.

(E) The CoREST ternary complex was cross-linked with increasing concentrations of CBDPSS.

(F) Schematic showing the CBDPSS cross-links identified in the CoREST ternary complex using mass spectrometry. Dotted lines indicated the cross-linked residues with the xQuest ID score above 14.5.

(G) The SAXS-derived model of the CoREST ternary complex with cross-linked residues highlighted with dotted lines in pink. Black arrows indicate the active sites of HDAC1 and LSD1.

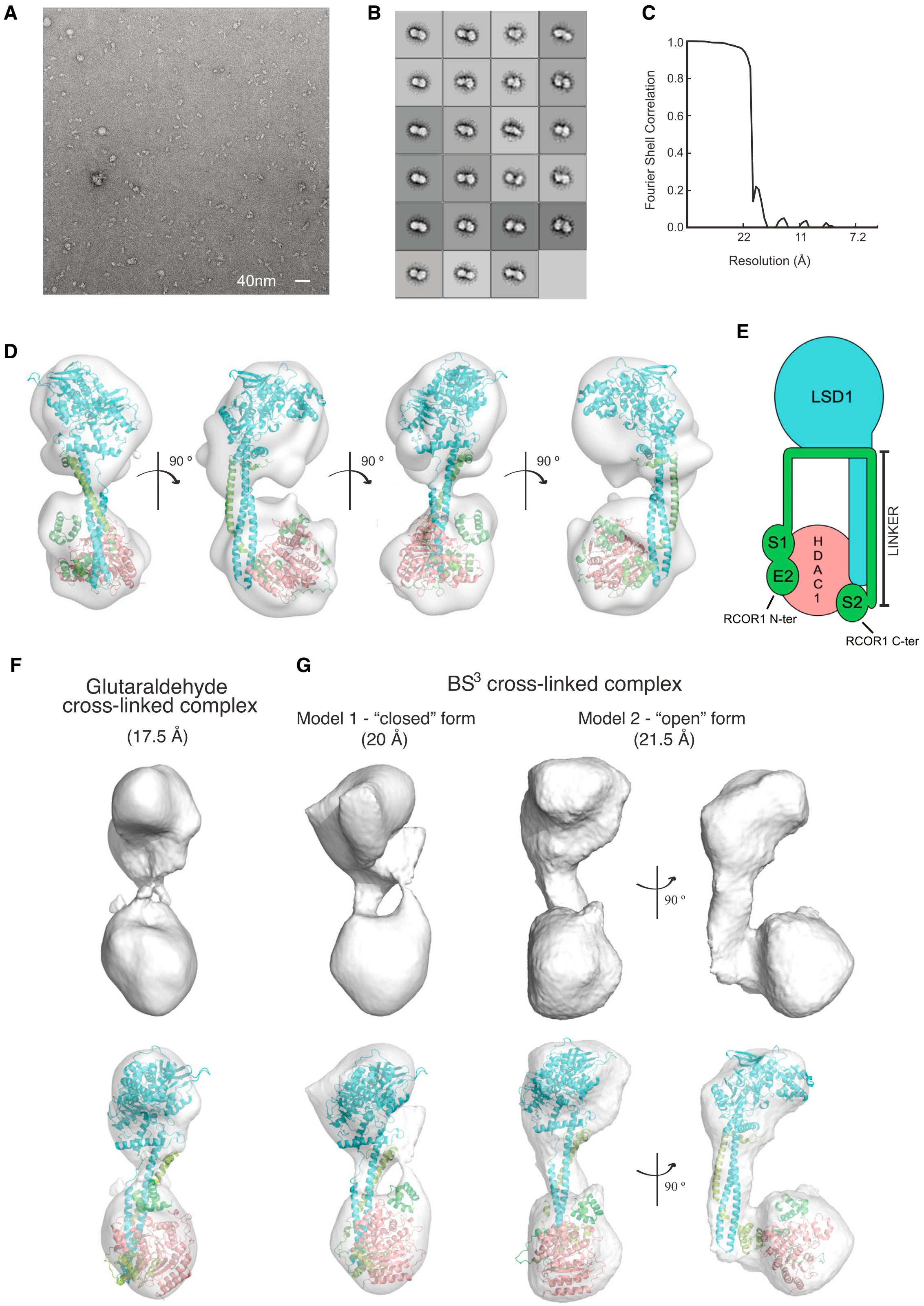

(legend on next page)

was designed to stabilize binding of the nucleosome to the FAD (flavin adenine dinucleotide) at the LSD1 active site (Culhane et al., 2006).

The complex between the 185-bp H3K4-propargyl nucleosomes and the holo CoREST complex was prepared at various sample ratios (Figure 5A). The holo CoREST complex forms both 1:1 and 2:1 bound complexes with the 185-bp H3K4-propargyl nucleosomes. However, a percentage of unbound nucleosome was always observed, even on adding excess CoREST. In order to reduce the heterogeneity of the sample, we performed a further purification step through a 2.4 mL Superdex 200 column (3.2/300). The fraction containing the largest proportion of CoREST:nucleosome 1:1 complex was selected and cross-linked with glutaraldehyde to make a sample for negative stain EM (Figure S7). Attempts to purify a CoREST:nucleosome 2:1 complex were not successful due to too much heterogeneity.

The micrographs of the predominantly 1:1 complex gave rise to rather heterogeneous 2D class averages. However, the majority of particles fell into a limited number of highly populated classes (Figure 5B). These clearly contain the familiar bi-lobed particles of the CoREST complex bound to a larger particle that resembles the known structure of a nucleosome. Using these highly populated class averages, it was possible to generate a 3D structural model of the complex with a nominal resolution of 26 Å (Figures 5C and 5D). The bi-lobed density of the CoREST complex strongly resembled the previous compact models observed in both the negative stain and cryo-EM grids. We were readily able to dock the known structures into the envelope (Figure 5E). It is clear that LSD1 within the CoREST complex is bound to the nucleosome (Luger et al., 1997; PDB: 1AOI) in such a way that the H3K4-propargyl group is able to engage with the active site of the LSD1 demethylase. When LSD1 is engaged with the substrate, the HDAC1 is rather far from the nucleosome and may not be able to target histone tails in the same nucleosome. Interestingly, positively charged residues in LSD1 are positioned such that they can mediate interaction with the phosphate backbone of the DNA around the dyad. Furthermore, the positively charged region that has been previously found to interact with RNA is ideally positioned to interact with the emerging linker DNA (Hirschi et al., 2016; Figures 5F and 5G).

DISCUSSION

We have expressed and purified a stable ternary CoREST complex in mammalian HEK293F cells. The complex is monomeric, and both enzymes in the complex are active, demonstrating that the complex is fully functional. In previous studies, we have used this complex to characterize a combined deacetylase and demethylase inhibitor and to investigate specificity of the complex for different modifications on a reconstituted nucleosome substrate (Kalin et al., 2018; Wu et al., 2018). In these studies, we noticed that the demethylase activity did not appear to follow Michaelis-Menten kinetics and therefore established a simultaneous, real-time demethylase and deacetylase assay. Using this approach, we have shown that the two enzymes within the CoREST complex, HDAC1 and LSD1, are coupled and mutually influence the activity of the other enzyme. Consistent with this, we showed that inhibitors and activators of one enzyme also influence the activity of the other enzyme. Indeed, the effects of $InsP_6$ are greater on the demethylase than the deacetylase reaction. Furthermore, we have found that both enzymes exist in at least two distinguishable states that differ in their kinetic properties. It is likely that this reflects two structurally distinct states of the complex. We also observed that the complex can only engage with one substrate at a time. However, a limitation of this study is that isolated peptides were used for the ^1H NMR-based assays rather than nucleosomes, because nucleosomes will impose spatial restraints that could influence the accessibility of the H3 tails.

To date, there have been no structural studies of the CoREST ternary complex. However, there have been numerous crystallographic studies of the LSD1:RCOR1 complex as well as modeling to predict the mode of interaction with nucleosomes (Baron et al., 2011; Baron and Vellore, 2012a, 2012b; Forneris et al., 2007; Pilotto et al., 2015; Yang et al., 2007, 2006). Although there is no structure of HDAC1 in complex with RCOR1, the structure of HDAC1:MTA1 complex (Millard et al., 2013; Watson et al., 2016) enables us to model the HDAC1:RCOR1 complex.

To investigate the physical relationship between the HDAC1 and LSD1 in the ternary complex, we have used SAXS, cross-linking-MS, negative-stain EM, and cryo-EM. The results from these complementary approaches are consistent with each other and reveal a bi-lobed structure with the LSD1 demethylase at one end and the HDAC1 at the other. The known LSD1:RCOR1 and HDAC1 structures fit well within the SAXS and EM envelopes. The orientation of the LSD1 is relatively well defined by the distinctive tower domain. In contrast, the orientation of the HDAC1 is less well defined and the calculated best fit to each of the EM envelopes is somewhat variable. In all the structures, HDAC1 is positioned close to the SANT2 domain of RCOR1 and likely makes a direct interaction with this domain. Previous models of the LSD1:RCOR1 in complex with a nucleosome have suggested that the SANT2 domain might bind to the outer surface of the DNA wrapped around the nucleosome

Figure 4. Structural Models of the CoREST Complex Generated Using Negative Stain and Cryo-electron Microscopy

(A) Electron micrograph of negatively stained CoREST complex.

(B) Reference-free 2D class averages of the particles used to generate the 3D model.

(C) Fourier shell correlation plot (CryoSparc). The resolution of the EM model was approximately 18 Å.

(D) Different views of the EM model of the CoREST complex (gray) fitted with the crystal structures of the MTA1:HDAC1 and LSD1:RCOR1 complexes. LSD1 is shown in cyan, HDAC1 in salmon, and MTA1/RCOR1 in green.

(E) Schematic representation of the structural arrangement of the CoREST complex. RCOR1 is green. The ELM2, SANT1, and SANT2 domains are indicated E2, S1, and S2, respectively.

(F) Refined 3D model of the glutaraldehyde cross-linked CoREST complex. The estimated resolution is 17.5 Å (Relion).

(G) Two different structural models were generated for the BS^3 cross-linked CoREST complex: a closed form (20 Å), which closely matches the glutaraldehyde cross-linked complex, and an open form (21.5 Å), in which the HDAC1 is pivoted away from the LSD1 tower domain.

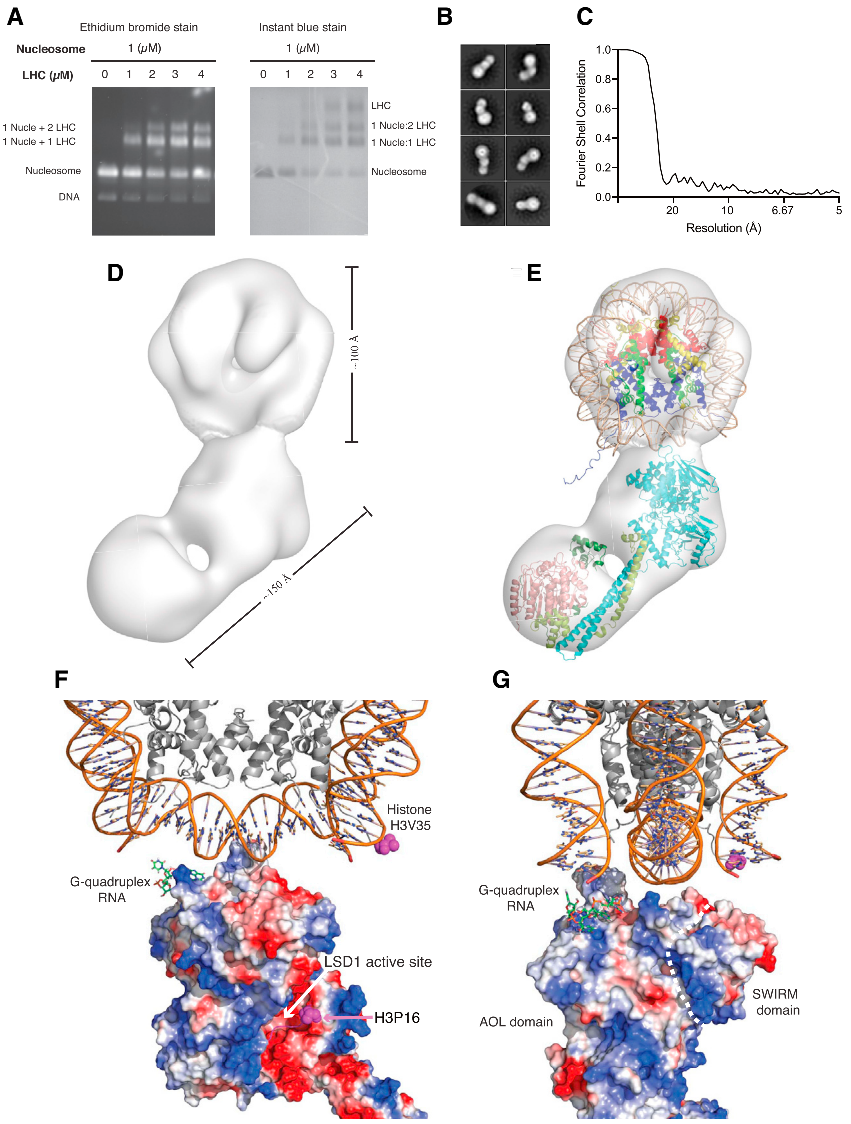

Figure 5. Structural Model of the CoREST Complex Bound to Semi-synthetic H3K4-Propargyl-Modified Nucleosome (Negative-Stain EM)

(A) Electrophoretic mobility shift assay (EMSA) assay of the CoREST complex (labeled LHC) binding to 185-bp H3K4-propargyl nucleosome.

(B) Reference-free 2D class averages of the particles used to generate the 3D model.

(C) Fourier shell correlation plot (Relion3.0). The resolution of the EM model was approximately 26 Å.

(D) 3D envelope of the CoREST complex bound to the nucleosome.

(E) Crystal structures of the nucleosome, HDAC1, LSD1, and RCOR1 fitted into the EM envelope. H3, blue; H4, dark green; H2A, yellow; H2B, red (PDB: 1AOI; Luger et al., 1997).

(F and G) Orthogonal views of the charged surface of the LSD1:RCOR positioned as seen in the EM structure of the CoREST complex bound to a nucleosome. The binding surface for RNA (nucleic acid) is highlighted as is the LSD1 active site with the H3 peptide (1–16 aas) shown in purple (PDB: 2V1D, Forneris et al., 2007; PDB: 4XBF, Hirschi et al., 2016).

(Pilotto et al., 2015; Yang et al., 2006). This has led to the concept of a nanoscale clamp positioning the LSD1:RCOR1 around the nucleosome (Baron and Vellore, 2012a, 2012b). The position of HDAC1 we observe in the ternary complex shows that it is in close proximity to the SANT2 domain. This suggests that it is unlikely that the ternary complex would be able to interact with a nucleosome in the way that has been predicted.

It is interesting that, in the BS^3 cross-linked cryo-EM sample, we observe a second (less populated) form of the complex. In this complex, the HDAC1 appears to be rotated away from the LSD1 TOWER domain. Although it is not possible to rule out that this is an artifact of the grid preparation and flash freezing, it may reflect an alternative form of the complex. It certainly seems likely that the alternative state of the complex revealed from the enzyme kinetics results from some structural change in the complex. Interestingly, molecular dynamics analyses of the LSD1:RCOR1 complex have suggested that the SANT2 domain may rotate and swing with respect to the TOWER of LSD1 (Baron and Vellore, 2012a, 2012b). This is consistent with the motion that would be required to create the alternative conformation under the BS^3 cross-linking conditions.

Given that that the structure we observe for the ternary CoREST complex appears inconsistent with previous models of how LSD1:RCOR1 binds nucleosomes, we wished to investigate how the ternary CoREST complex interacts with a mono-nucleosome using negative-stain EM. To aid formation of a stable complex, we prepared a modified nucleosome in which H3K4 was replaced with a propargylamine group that can form a non-reversible coupling with the FAD co-factor in the active site of LSD1. We reasoned that the resulting complex would provide an experimental understanding of how LSD1, in the context of the ternary CoREST complex, targets methylated K4 in a chromatin substrate.

Although the 2D class averages revealed some heterogeneity in the complex, a dominant proportion of particles were grouped into a small consistent set of class averages, in which the bi-lobed CoREST complex was clearly docked with the edge of the nucleosome disk. The HDAC1 is rather distant from the nucleosome and unlikely to be able to interact with histone tails from the same nucleosome. It may be possible for HDAC1 to target an adjacent nucleosome; however, the enzymology suggests that only one substrate can be engaged at a time. The LSD1 is positioned such that a relatively basic surface of the demethylase domain of LSD1 (but not the SWIRM domain) interacts with the

nucleosomal DNA close to the dyad of the nucleosome, where the linker DNA enters and leaves the nucleosome. The H3K4-propargyl in the histone tail can readily reach the active site of LSD1. Intriguingly, the region of LSD1 that has previously been shown to interact with RNA (Hirschi et al., 2016) is positioned very close to where the linker DNA would likely interact with the LSD1 demethylase domain. It is not clear what the implications of this might be. However, two studies have shown that CoREST preferentially binds nucleosomes with longer linker DNA (Kim et al., 2015; Wu et al., 2018). This finding prompted us to use 185-bp DNA in this structure. Unfortunately, the linker DNA is not visible at this resolution, and a more detailed understanding of this will require a higher resolution structure.

In conclusion, our investigation into the CoREST ternary complex has revealed a molecular machine in which the two enzymes, deacetylase and demethylase, are functionally and structurally coupled.

STAR★METHODS

Detailed methods are provided in the online version of this paper and include the following:

- KEY RESOURCES TABLE
- LEAD CONTACT AND MATERIALS AVAILABILITY
- EXPERIMENTAL MODEL AND SUBJECT DETAILS
  - Mammalian Protein Expression
- METHOD DETAILS
  - Mammalian Protein Purification
  - Size-Exclusion Chromatography with Multi-Angle Light Scattering (SEC-MALS)
  - Boc-Lys(Ac)-AMC HDAC Assay
  - Horse Radish Peroxide (HRP)-coupled Demethylase Assay
  - Peptide synthesis for NMR based enzymatic assay
  - 1D-NMR based Real-time Enzymatic Assay
  - Analysis of NMR spectra
  - Data analysis of NMR based enzymatic assay
  - Small-Angle Light Scattering (SAXS)
  - Chemical cross-linking mass spectrometry
  - Histone expression and purification
  - Preparation of propargylamine-containing mimic of dimethyl Lys4 histone H3
  - Histone octamer refolding and nucleosome reconstitution
  - Structural determination of the CoREST complex using negative stain EM
  - Cryo Electron Microscopy sample preparation and imaging
- QUANTIFICATION AND STATISTICAL ANALYSIS
  - Experimental Replicates and Quantification
  - Statistical Analysis
- DATA AND CODE AVAILABILITY

SUPPLEMENTAL INFORMATION

Supplemental Information can be found online at https://doi.org/10.1016/j.celrep.2020.01.091.

ACKNOWLEDGMENTS

We are grateful to the PROTEX facility (University of Leicester) for preparation of expression clones; the staff at B21 at the Diamond Light Source for the SAXS data collection; Ian Hands-Portman at the Warwick Life Sciences Imaging Suite (now Advanced Bioimaging Research Technology Platform), University of Warwick, using equipment funded by the Wellcome Trust (055663/Z/98/Z); and the eBIC facility at the Diamond Light Source for initial cryo-EM datasets; and the MRC Toxicology Electron Microscopy facility. We acknowledge the Midlands Regional CryoEM Facility at the Leicester Institute of Structural and Chemical Biology (LISCB) and major funding from MRC (MC_PC_17136). J.W.R.S. is a Wellcome Trust Senior Investigator (100237/Z/12/Z) and Royal Society Wolfson Research Merit Award Holder. D.F.H. was supported by the Wellcome Trust (101569/Z/13/Z) and Leverhulme Trust (RPG-2016-268). P.A.C. was supported by the NIH (GM62437).

AUTHOR CONTRIBUTIONS

The overall project was conceived by Y.S., L.F., and J.W.R.S. Construct design, protein expression, purification, fluorogenic enzyme assays, SAXS, XL-MS, and EM sample preparation were performed by Y.S. The NMR assay was designed and interpreted by Y.S., D.F.H., and J.W.R.S. NMR data were collected and analyzed by Y.S., L.D., M.B.A.K., and D.F.H. Modified histone tail peptides were prepared by N.R. and A.G.J. The H3K4-propargyl nucleosome was designed and prepared by M.W. and P.A.C. Negative-stain EM data were collected by N.M. and A.S. Cryo-EM data were collected by Y.S. and C.G.S. EM data were analyzed and interpreted by Y.S., L.F., T.J.R., and J.W.R.S. The manuscript was drafted by Y.S., D.F.H., and J.W.R.S. and critically commented on by all authors.

DECLARATION OF INTERESTS

The authors declare no competing interests.

Received: September 13, 2019
Revised: December 31, 2019
Accepted: January 24, 2020
Published: February 25, 2020

REFERENCES

Abramowitz, M., and Stegun, I.A. (1965). Handbook of Mathematical Functions (US Department of Commerce).

Andrés, M.E., Burger, C., Peral-Rubio, M.J., Battaglioli, E., Anderson, M.E., Grimes, J., Dallman, J., Ballas, N., and Mandel, G. (1999). CoREST: a functional corepressor required for regulation of neural-specific gene expression. Proc. Natl. Acad. Sci. USA *96*, 9873–9878.

Bader, G., and Deuflhard, P. (1983). A semi-implicit mid-point rule for stiff systems of ordinary differential equations. Numer. Math. *41*, 373–398.

Ballas, N., Battaglioli, E., Atouf, F., Andrés, M.E., Chenoweth, J., Anderson, M.E., Burger, C., Moniwa, M., Davie, J.R., Bowers, W.J., et al. (2001). Regulation of neuronal traits by a novel transcriptional complex. Neuron *31*, 353–365.

Baron, R., and Vellore, N.A. (2012a). LSD1/CoREST reversible opening-closing dynamics: discovery of a nanoscale clamp for chromatin and protein binding. Biochemistry *51*, 3151–3153.

Baron, R., and Vellore, N.A. (2012b). LSD1/CoREST is an allosteric nanoscale clamp regulated by H3-histone-tail molecular recognition. Proc. Natl. Acad. Sci. USA *109*, 12509–12514.

Baron, R., Binda, C., Tortorici, M., McCammon, J.A., and Mattevi, A. (2011). Molecular mimicry and ligand recognition in binding and catalysis by the histone demethylase LSD1-CoREST complex. Structure *19*, 212–220.

Culhane, J.C., Szewczuk, L.M., Liu, X., Da, G., Marmorstein, R., and Cole, P.A. (2006). A mechanism-based inactivator for histone demethylase LSD1. J. Am. Chem. Soc. *128*, 4536–4537.

Delaglio, F., Grzesiek, S., Vuister, G.W., Zhu, G., Pfeifer, J., and Bax, A. (1995). NMRPipe: a multidimensional spectral processing system based on UNIX pipes. J. Biomol. NMR *6*, 277–293.

Delcuve, G.P., Khan, D.H., and Davie, J.R. (2012). Roles of histone deacetylases in epigenetic regulation: emerging paradigms from studies with inhibitors. Clin. Epigenetics *4*, 5–13.

Efron, B., and Tibshirani, R. (1986). Bootstrap methods for standard errors, confidence intervals, and other measures of statistical accuracy. Stat. Sci. *1*, 54–75.

Forneris, F., Binda, C., Vanoni, M.A., Battaglioli, E., and Mattevi, A. (2005). Human histone demethylase LSD1 reads the histone code. J. Biol. Chem. *280*, 41360–41365.

Forneris, F., Binda, C., Adamo, A., Battaglioli, E., and Mattevi, A. (2007). Structural basis of LSD1-CoREST selectivity in histone H3 recognition. J. Biol. Chem. *282*, 20070–20074.

Foster, C.T., Dovey, O.M., Lezina, L., Luo, J.L., Gant, T.W., Barlev, N., Bradley, A., and Cowley, S.M. (2010). Lysine-specific demethylase 1 regulates the embryonic transcriptome and CoREST stability. Mol. Cell. Biol. *30*, 4851–4863.

Hansen, D.F., Yang, D., Feng, H., Zhou, Z., Wiesner, S., Bai, Y., and Kay, L.E. (2007). An exchange-free measure of 15N transverse relaxation: an NMR spectroscopy application to the study of a folding intermediate with pervasive chemical exchange. J. Am. Chem. Soc. *129*, 11468–11479.

Hesham, H.M., Lasheen, D.S., and Abouzid, K.A.M. (2018). Chimeric HDAC inhibitors: comprehensive review on the HDAC-based strategies developed to combat cancer. Med. Res. Rev. *38*, 2058–2109.

Hirschi, A., Martin, W.J., Luka, Z., Loukachevitch, L.V., and Reiter, N.J. (2016). G-quadruplex RNA binding and recognition by the lysine-specific histone demethylase-1 enzyme. RNA *22*, 1250–1260.

Humphrey, G.W., Wang, Y., Russanova, V.R., Hirai, T., Qin, J., Nakatani, Y., and Howard, B.H. (2001). Stable histone deacetylase complexes distinguished by the presence of SANT domain proteins CoREST/kiaa0071 and Mta-L1. J. Biol. Chem. *276*, 6817–6824.

Hura, G.L., Menon, A.L., Hammel, M., Rambo, R.P., Poole, F.L., 2nd, Tsutakawa, S.E., Jenney, F.E., Jr., Classen, S., Frankel, K.A., Hopkins, R.C., et al. (2009). Robust, high-throughput solution structural analyses by small angle X-ray scattering (SAXS). Nat. Methods *6*, 606–612.

Itoh, T., Fairall, L., Muskett, F.W., Milano, C.P., Watson, P.J., Arnaudo, N., Saleh, A., Millard, C.J., El-Mezgueldi, M., Martino, F., and Schwabe, J.W.R. (2015). Structural and functional characterization of a cell cycle associated HDAC1/2 complex reveals the structural basis for complex assembly and nucleosome targeting. Nucleic Acids Res. *43*, 2033–2044.

Kalin, J.H., Wu, M., Gomez, A.V., Song, Y., Das, J., Hayward, D., Adejola, N., Wu, M., Panova, I., Chung, H.J., et al. (2018). Targeting the CoREST complex with dual histone deacetylase and demethylase inhibitors. Nat. Commun. *9*, 53.

Kim, S.-A., Chatterjee, N., Jennings, M.J., Bartholomew, B., and Tan, S. (2015). Extranucleosomal DNA enhances the activity of the LSD1/CoREST histone demethylase complex. Nucleic Acids Res. *43*, 4868–4880.

Lakowski, B., Roelens, I., and Jacob, S. (2006). CoREST-like complexes regulate chromatin modification and neuronal gene expression. J. Mol. Neurosci. *29*, 227–239.

Lee, M.G., Wynder, C., Cooch, N., and Shiekhattar, R. (2005). An essential role for CoREST in nucleosomal histone 3 lysine 4 demethylation. Nature *437*, 432–435.

Lee, M.G., Wynder, C., Bochar, D.A., Hakimi, M.-A., Cooch, N., and Shiekhattar, R. (2006). Functional interplay between histone demethylase and deacetylase enzymes. Mol. Cell. Biol. *26*, 6395–6402.

Leitner, A., Walzthoeni, T., and Aebersold, R. (2014). Lysine-specific chemical cross-linking of protein complexes and identification of cross-linking sites using LC-MS/MS and the xQuest/xProphet software pipeline. Nat. Protoc. *9*, 120–137.

Lowary, P.T., and Widom, J. (1998). New DNA sequence rules for high affinity binding to histone octamer and sequence-directed nucleosome positioning. J. Mol. Biol. *276*, 19–42.

Luger, K., Mäder, A.W., Richmond, R.K., Sargent, D.F., and Richmond, T.J. (1997). Crystal structure of the nucleosome core particle at 2.8 A resolution. Nature *389*, 251–260.

Millard, C.J., Watson, P.J., Celardo, I., Gordiyenko, Y., Cowley, S.M., Robinson, C.V., Fairall, L., and Schwabe, J.W.R. (2013). Class I HDACs share a common mechanism of regulation by inositol phosphates. Mol. Cell *51*, 57–67.

Millard, C.J., Watson, P.J., Fairall, L., and Schwabe, J.W.R. (2017). Targeting class I histone deacetylases in a "complex" environment. Trends Pharmacol. Sci. *38*, 363–377.

Oberoi, J., Fairall, L., Watson, P.J., Yang, J.-C., Czimmerer, Z., Kampmann, T., Goult, B.T., Greenwood, J.A., Gooch, J.T., Kallenberger, B.C., et al. (2011). Structural basis for the assembly of the SMRT/NCoR core transcriptional repression machinery. Nat. Struct. Mol. Biol. *18*, 177–184.

Petoukhov, M.V., Franke, D., Shkumatov, A.V., Tria, G., Kikhney, A.G., Gajda, M., Gorba, C., Mertens, H.D.T., Konarev, P.V., and Svergun, D.I. (2012). New developments in the *ATSAS* program package for small-angle scattering data analysis. J. Appl. Cryst. *45*, 342–350.

Pettersen, E.F., Goddard, T.D., Huang, C.C., Couch, G.S., Greenblatt, D.M., Meng, E.C., and Ferrin, T.E. (2004). UCSF Chimera–a visualization system for exploratory research and analysis. J. Comput. Chem. *25*, 1605–1612.

Piiadov, V., Ares de Araújo, E., Oliveira Neto, M., Craievich, A.F., and Polikarpov, I. (2019). SAXSMoW 2.0: Online calculator of the molecular weight of proteins in dilute solution from experimental SAXS data measured on a relative scale. Protein Sci. *28*, 454–463.

Pilotto, S., Speranzini, V., Tortorici, M., Durand, D., Fish, A., Valente, S., Forneris, F., Mai, A., Sixma, T.K., Vachette, P., and Mattevi, A. (2015). Interplay among nucleosomal DNA, histone tails, and corepressor CoREST underlies LSD1-mediated H3 demethylation. Proc. Natl. Acad. Sci. USA *112*, 2752–2757.

Press, W.H., Teukolsky, S.A., Vetterling, W.T., and Flannery, B.P. (1992). Numerical Recipes in C: the Art of Scientific Computing (Cambridge University Press).

Punjani, A., Rubinstein, J.L., Fleet, D.J., and Brubaker, M.A. (2017). cryoSPARC: algorithms for rapid unsupervised cryo-EM structure determination. Nat. Methods *14*, 290–296.

Rowe, E.M., Xing, V., and Biggar, K.K. (2019). Lysine methylation: Implications in neurodegenerative disease. Brain Res. *1707*, 164–171.

Saleque, S., Kim, J., Rooke, H.M., and Orkin, S.H. (2007). Epigenetic regulation of hematopoietic differentiation by Gfi-1 and Gfi-1b is mediated by the cofactors CoREST and LSD1. Mol. Cell *27*, 562–572.

Scheres, S.H.W. (2012). RELION: implementation of a Bayesian approach to cryo-EM structure determination. J. Struct. Biol. *180*, 519–530.

Shi, Y., Lan, F., Matson, C., Mulligan, P., Whetstine, J.R., Cole, P.A., Casero, R.A., and Shi, Y. (2004). Histone demethylation mediated by the nuclear amine oxidase homolog LSD1. Cell *119*, 941–953.

Shi, Y.-J., Matson, C., Lan, F., Iwase, S., Baba, T., and Shi, Y. (2005). Regulation of LSD1 histone demethylase activity by its associated factors. Mol. Cell *19*, 857–864.

Svergun, D., Barberato, C., and Koch, M.H.J. (1995). CRYSOL - a program to evaluate X-ray solution scattering of biological macromolecules from atomic coordinates. J. Appl. Cryst. *28*, 768–773.

Tang, G., Peng, L., Baldwin, P.R., Mann, D.S., Jiang, W., Rees, I., and Ludtke, S.J. (2007). EMAN2: an extensible image processing suite for electron microscopy. J. Struct. Biol. *157*, 38–46.

Wagner, T., Merino, F., Stabrin, M., Moriya, T., Antoni, C., Apelbaum, A., Hagel, P., Sitsel, O., Raisch, T., Prumbaum, D., et al. (2019). SPHIRE-crYOLO is a fast and accurate fully automated particle picker for cryo-EM. Commun. Biol *2*, 218.

Wang, J., Scully, K., Zhu, X., Cai, L., Zhang, J., Prefontaine, G.G., Krones, A., Ohgi, K.A., Zhu, P., Garcia-Bassets, I., et al. (2007). Opposing LSD1

complexes function in developmental gene activation and repression programmes. Nature *446*, 882–887.

Watson, P.J., Fairall, L., Santos, G.M., and Schwabe, J.W.R. (2012). Structure of HDAC3 bound to co-repressor and inositol tetraphosphate. Nature *481*, 335–340.

Watson, P.J., Millard, C.J., Riley, A.M., Robertson, N.S., Wright, L.C., Godage, H.Y., Cowley, S.M., Jamieson, A.G., Potter, B.V.L., and Schwabe, J.W.R. (2016). Insights into the activation mechanism of class I HDAC complexes by inositol phosphates. Nat. Commun. *7*, 11262.

Wu, M., Hayward, D., Kalin, J.H., Song, Y., Schwabe, J.W., and Cole, P.A. (2018). Lysine-14 acetylation of histone H3 in chromatin confers resistance to the deacetylase and demethylase activities of an epigenetic silencing complex. eLife *7*, 384.

Yang, M., Gocke, C.B., Luo, X., Borek, D., Tomchick, D.R., Machius, M., Otwinowski, Z., and Yu, H. (2006). Structural basis for CoREST-dependent demethylation of nucleosomes by the human LSD1 histone demethylase. Mol. Cell *23*, 377–387.

Yang, M., Culhane, J.C., Szewczuk, L.M., Gocke, C.B., Brautigam, C.A., Tomchick, D.R., Machius, M., Cole, P.A., and Yu, H. (2007). Structural basis of histone demethylation by LSD1 revealed by suicide inactivation. Nat. Struct. Mol. Biol. *14*, 535–539.

You, A., Tong, J.K., Grozinger, C.M., and Schreiber, S.L. (2001). CoREST is an integral component of the CoREST- human histone deacetylase complex. Proc. Natl. Acad. Sci. USA *98*, 1454–1458.

Zhang, K. (2016). Gctf: Real-time CTF determination and correction. J. Struct. Biol. *193*, 1–12.

Zheng, S.Q., Palovcak, E., Armache, J.-P., Verba, K.A., Cheng, Y., and Agard, D.A. (2017). MotionCor2: anisotropic correction of beam-induced motion for improved cryo-electron microscopy. Nat. Methods *14*, 331–332.

Zhu, A., Romero, R., and Petty, H.R. (2010). Amplex UltraRed enhances the sensitivity of fluorimetric pyruvate detection. Anal. Biochem. *403*, 123–125.

Zivanov, J., Nakane, T., Forsberg, B.O., Kimanius, D., Hagen, W.J., Lindahl, E., and Scheres, S.H. (2018). New tools for automated high-resolution cryo-EM structure determination in RELION-3. eLife *7*, e42166.

STAR★METHODS

KEY RESOURCES TABLE

REAGENT or RESOURCE	SOURCE	IDENTIFIER
Chemicals, Peptides, and Recombinant Proteins		
Polyethylenimine (PEI)	Sigma-Aldrich	CAT# 408727
Anti-Flag M2 affinity gel	Sigma-Aldrich	CAT# A2220
Rink Amide SpheriTide resin (1.05 mmol/g substitution)	CEM	CAT# R002-A
Fmoc-Ala-OH	Novabiochem	CAT# 852003 CAS# 35661-39-3
Fmoc-Arg(Pbf)-OH	Novabiochem	CAT# 852067 CAS# 154445-77-9
Fmoc-Thr(^{t}Bu)-OH	Novabiochem	CAT# 852000 CAS# 71989-35-0
Fmoc-Lys(Me,Boc)-OH	Novabiochem	CAT# 852106 CAS# 951695-85-5
Fmoc-Gln(Trt)-OH	Novabiochem	CAT# 852045 CAS# 132327-80-1
Fmoc-Lys(Boc)-OH	Novabiochem	CAT# 852012 CAS# 71989-26-9
Fmoc-Ser(tBu)OH	Novabiochem	CAT# 852019 CAS# 71989-33-8
Fmoc-Gly-OH	Novabiochem	CAT# 852001 CAS# 29022-11-5
Fmoc-Pro-OH	Novabiochem	CAT# 852017 CAS# 71989-31-6
Fmoc-Leu-OH	Novabiochem	CAT# 852011 CAS# 35661-60-0
Fmoc-Lys(Ac)-OH	Novabiochem	CAT# 852042 CAS# 159766-56-0
N,*N*-dimethylformaldehyde (DMF)	Rathburn Chemicals	CAT# PTS6020 CAS# 68-12-2
1-Methyl 2-pyrrolidone (NMP)	Rathburn Chemicals	CAT# PTS6035 CAS# 872-50-4
Piperidine	Sigma-Aldrich	CAT# 104094 CAS# 110-89-4
O-(1H-6-Chlorobenzotriazole-1-yl)-1,1,3,3-tetramethyluronium hexafluorophosphate (HCTU)	Novabiochem	CAT# 851012 CAS# 330645-87-9
OxymaPure	Novabiochem	CAT# 8.51086 CAS# 3849-21-6
Diisopropylethylamine (DIPEA)	Sigma-Aldrich	CAT# D125806 CAS# 7087-68-5
Trifluoroacetic acid (TFA)	Sigma-Aldrich	CAT# T6508 CAS# 76-05-1
Triethylsilane (TES)	Sigma-Aldrich	CAT# 230197 CAS# 617-86-7
Fmoc-Gln(Trt)-Thr(ψMe,Mepro)-OH	Novabiochem	CAT# 8.52198 CAS# 1572725-72-4
Fmoc-Ser(tBu)-Thr(ψMe,Mepro)-OH	Novabiochem	CAT# 8.52192CAS# 1266350-99-5
MS-275 (Entinostat, SNDX-275)	Sigma-Aldrich	CAT# EPS002 CAS# 209783-80-2
TSA (Trichostatin A)	Sigma-Aldrich	CAT# T8552 CAS# 58880-19-6
SAHA (Vorinostat)	Sigma-Aldrich	CAT# SML0061 CAS# 149647-78-9
2-PCPA (*trans*-2-Phenylcyclopropylamine hydrochloride)	Sigma-Aldrich	CAT# P8511 CAS# 1986-47-6
$InsP_4$ (D-myo-Inositol-1,4,5,6-tetraphosphate)	Cayman Chemical	CAT# 10007783 CAS# 157542-47-7
$InsP_5$ (D-myo-Inositol-1,3,4,5,6-pentaphosphate)	Cayman Chemical	CAT# 10007784
$InsP_6$ (Phytic Acid)	Sigma-Aldrich	CAT# 68388 CAS# 14306-25-3
Boc-Lys(ac)-AMC	BaChem	CAT# 4033972
Horseradish peroxidase (HRP), EIA grade	ThermoFisher Scientific	CAT# 012001
Amplex Red Reagent	ThermoFisher Scientific	CAT# A22177
CBDPSS	Creative Molecules Inc.	CAT# 014SS
Glutaraldehyde	Sigma-Aldrich	CAT# G5882 CAS# 111-20-8
BS^3 (Sulfo-DSS) Crosslinker	ThermoFisher Scientific	CAT# A39266
2-(Methylamino)ethanol	Sigma-Aldrich	CAT# 471445 CAS# 109-83-1
Propargyl bromide	Sigma-Aldrich	CAT# 81831 CAS# 106-96-7
Histone proteins	(Wu et al., 2018)	N/A
H3K4me 1-21 peptide	This paper	N/A
H3K9ac 1-21 peptide	This paper	N/A
H3K4meK9ac 1-21 peptide	This paper	N/A

(*Continued on next page*)

Continued

REAGENT or RESOURCE	SOURCE	IDENTIFIER
Deposited Data		
Negative stain map: CoREST complex	EMDB	EMDB: EMD-10626
Cryo-EM map: Glutaraldehyde crosslinked complex	EMDB	EMDB: EMD-10627
Cryo-EM map: BS3 crosslinked complex-closed form	EMDB	EMDB: EMD-10628
Cryo-EM map: BS3 crosslinked complex-open form	EMDB	EMDB: EMD-10629
Negative stain map: CoREST-nucleosome complex	EMDB	EMDB: EMD-10630
SAXS data	SASDB	SASDB: SASDH45
Experimental Models: Cell Lines		
FreeStyle 293-F cells	ThermoFisher Scientific	CAT# R79007
Oligonucleotides		
5′ Primer for cloning CoREST (aa 86) protein into pLEICS12 vector: GTATTTTCAGGGCGCCATGTG GGAGGAAGGCAGC	Eurofins Genomics	N/A
3′ Primer for cloning CoREST protein (aa 485) into pLEICS12 vector: GACGGAGCTCGAATTTCAGG AGGCAGATGCATATCT	Eurofins Genomics	N/A
5′ Primer for cloning LSD1 protein (aa 1) into pLEICS12 vector: ACCCAAGCTTGGTACCATGTT ATCTGGGAAGAAGGCG	Eurofins Genomics	N/A
3′ Primer for cloning LSD1 protein (aa 852) into pLEICS12 vector: GACGGAGCTCGAATTTCACA TGCTTGGGGACTGCTGTGC	Eurofins Genomics	N/A
5′ Primer for the mutagenesis of H3(K4C): CTCGTACTTGTCAGACCGCCCGCAAG	IDT	N/A
3′ Primer for the mutagenesis of H3(K4C): GGCGGTCTGACAAGTACGAGCCATATG	IDT	N/A
185 bp 601 DNA	(Wu et al., 2018)	N/A
Recombinant DNA		
IMAGE clone: CoREST	Source BioScience	ID: 40080558
IMAGE clone: LSD1	Source BioScience	ID: 5298150
Plasmid: pLEICS12-$(His)_{10}(Flag)_3$-CoREST(aa 86-485)	This paper	N/A
Plasmid: pLEICS12-LSD1 (full length)	This paper	N/A
Plasmid: pLEICS12-HDAC1 (full length)	(Millard et al., 2013)	N/A
Software and Algorithms		
Prism (version 7.0)	GraphPad	https://www.graphpad.com/scientific-software/prism/
NMRPipe	(Delaglio et al., 1995)	https://www.ibbr.umd.edu/nmrpipe/install.html
FuDA	(Hansen et al., 2007)	https://www.ucl.ac.uk/hansen-lab/
NMR data fitting algorithms	This paper	https://www.ucl.ac.uk/hansen-lab/
ScÅtter	(Hura et al., 2009)	http://www.bioisis.net; https://bl1231.als.lbl.gov/scatter
SAXS MoW	(Piiadov et al., 2019)	http://saxs.ifsc.usp.br/
CORAL	(Petoukhov et al., 2012)	https://www.embl-hamburg.de/biosaxs/atsas-online/coral.php
CRYSOL	(Svergun et al., 1995)	https://www.embl-hamburg.de/biosaxs/atsas-online/crysol.php
xQuest	(Leitner et al., 2014)	http://prottools.ethz.ch/orinner/public/htdocs/xquest/
EMAN2	(Tang et al., 2007)	https://blake.bcm.edu/emanwiki/EMAN2

(*Continued on next page*)

Continued

REAGENT or RESOURCE	SOURCE	IDENTIFIER
SPHIRE-crYOLO	(Wagner et al., 2019)	http://sphire.mpg.de/wiki/doku.php?id=pipeline:window:cryolo
Relion2	(Scheres, 2012)	https://www3.mrc-lmb.cam.ac.uk/relion/index.php/Main_Page
Relion3.0	(Zivanov et al., 2018)	
CryoSparc	(Punjani et al., 2017)	https://cryosparc.com/
Pymol (Version 1.8)	Schrödinger, LLC	https://pymol.org/2/
Chimera (Version 1.13.1)	(Pettersen et al., 2004)	https://www.cgl.ucsf.edu/chimera/

LEAD CONTACT AND MATERIALS AVAILABILITY

Further information and requests for resources and reagents should be directed to and will be fulfilled by the Lead Contact, John Schwabe (john.schwabe@le.ac.uk).

All unique reagents generated in this study are available from the lead contact with a completed Materials Transfer Agreement.

EXPERIMENTAL MODEL AND SUBJECT DETAILS

Mammalian Protein Expression

The CoREST ternary complex was comprised of full length LSD1 (UniProt ID: O60341), full length HDAC1 (UniProt ID: Q13547) and N-terminally truncated RCOR1 (86-485aa) (UniProt ID: Q9UKL0). The pcDNA3 vector was used to create plasmids encoding the different proteins. The RCOR1 constructs contained an N-terminal (His)10(Flag)3 tag followed by a Tev protease cleavage site. The constructs for ternary complex were co-transfected into suspension-grow HEK293F cells (ThermoFisher Scientific) with polyethylenimine (PEI) (Sigma) and harvested after 48 hours as described previously (Kalin et al., 2018; Wu et al., 2018).

METHOD DETAILS

Mammalian Protein Purification

Cells were lysed in buffer containing 50 mM Tris/Cl pH 7.5, 50 mM potassium acetate, 5% v/v glycerol, 0.4% v/v Triton X-100, and Roche Complete Protease Inhibitor (buffer A). Insoluble materials were removed by centrifugation. The complex was purified on Flag resin (Anti-Flag M2 affinity gel, Sigma). The resin was washed once with buffer A, three times with buffer B (50 mM Tris/Cl pH 7.5, 50 mM potassium acetate, and 5% v/v glycerol), and three times with buffer C (50 mM Tris pH 7.5, 50 mM potassium acetate, 5% v/v glycerol and 0.5 mM TCEP) followed by an overnight TEV protease cleavage in buffer C. The complex was further purified by gel filtration chromatography on a Superose 6 column (GE Healthcare) in buffer containing 25 mM Tris pH 7.5, 50 mM potassium acetate, and 0.5 mM TCEP.

Size-Exclusion Chromatography with Multi-Angle Light Scattering (SEC-MALS)

Purified CoREST ternary complex that has been gel filtrated was concentrated to > 1 mg/ml. The complex was reapplied to a Superose 6 column. The mass of the complex was detected on elution with an 18-angle MALS light scattering detector (Dawn® HELEOS® II) coupled with a differential Refractive Index detector (Optilab® T-rEX) (Wyatt Technology).

Boc-Lys(Ac)-AMC HDAC Assay

HDAC activity of the CoREST complex was measured using a fluorescent-based HDAC assay with Boc-Lys(Ac)-AMC substrate. 25 nM of purified complex, and 500 μM of substrate were used in a final volume of 50 μl in assay buffer (50 mM Tris pH 7.5, 50 mM NaCl, 0.1 mg/ml BSA). Inositol phosphates (100 μM) and HDAC inhibitors (5 μM) were tested for their ability to activate or repress the complex by pre-incubating with the complex in varying concentrations at 37°C for 30 minutes before adding the substrate. The assay was developed by the addition of 50 μl of developer solution (2 mM TSA, 10 mg/ml Trypsin, 50 mM Tris pH 7.5, 100 mM NaCl). Fluorescence was measured at 335/460 nm using a Victor X5 plate reader (Perkin Elmer). Data analyzed using GraphPad Prism (version 7.0, GraphPad Software, Inc.).

Horse Radish Peroxide (HRP)-coupled Demethylase Assay

The Demethylase assay was performed using an Amplex UltraRed reagent (Thermo Fisher Scientific) which fluoresces on a per-mole basis in a HRP-coupled assay (Zhu et al., 2010). A final concentration of 50 nM CoREST complex was used in a reaction volume of 100 μl. For LSD1 inhibition by LSD1 inhibitors, the protein sample was pre-incubated with the inhibitors for half hour in assay buffer containing 20 mM HEPES pH 7.5 and 50 mM NaCl at room temperature. Amplex UltraRed reagent (10 μM) and HRP (0.04 mg/ml)

were added before the assay was initiated with 15 μM of H3K4me peptide substrate. Fluorescence was measured at 530/590 nm using a Victor X5 plate reader (Perkin Elmer) after 30 minutes. All measurements were performed in triplicate and data analyzed using GraphPad Prism.

Peptide synthesis for NMR based enzymatic assay

The peptides were synthesized on a Biotage Initiator$^+$ Alstra machine on Rink Amide SpheriTide resin (1.05 mmol/g substitution, CEM). Fmoc-protected amino acids were made up as a solution of 0.2 M in DMF to give 5 equivalents relative to the resin once added to the reaction vessel. The activator was made up to 0.5 M HCTU in DMF and the activator base was made up to 2 M DIPEA in NMP. These solutions allowed for 5 equivalents of activator and 10 equivalents of activator base relative to the resin. Deprotection solution was made up to 20% v/v piperidine in DMF solution with 0.1M OxymaPure. Double coupling of Fmoc-Arg(Pbf)-OH was carried out at room temperature for 25 mins, then heated to 75°C for 5 mins, drained and a second coupling step carried out at 75°C for 5 mins. All other amino acids were coupled at 75°C for 5 mins. Deprotections were carried out at 75°C for 30 s, followed by a second deprotection at 75°C for 3 mins. Pseudoproline residues were used to help prevent the formation of on-resin secondary structures. Fmoc-Gln(Trt)-Thr(ψMe,Mepro)-OH for Gln5-Thr6 and Fmoc-Ser(tBu)-Thr(ψMe,Mepro)-OH for Ser11-Thr12.

Synthesized peptides were cleaved from the resin using a cleavage cocktail of TFA/TES/water (95:2.5:2.5) for 3 hours before being drained and the TFA blown off with a stream of nitrogen. The peptide was precipitated and washed three times in cold diethyl ether and spun down to a pellet before the diethyl ether removed and the peptide dried under a steady stream of nitrogen.

Crude peptides were purified by reverse-phase HPLC using a Dionex Ultimate 3000 system with a Phenomenex Gemini-NX 5 μm C18 110 Å AXIA packed column with dimensions 250 × 21.20 mm and purity confirmed by analytical reverse-phase HPLC using a Dionex Ultimate 3000 system with a Phenomenex Aeris 5 μm Peptide XB-C18 100 Å packed column with dimensions of 150 × 4.6 mm. LC-MS were run using a Xevo QTof mass spectrometer (Waters) coupled to an Acquity LC system (Waters) using an Acquity UPLC BEH C18 column (2.1 × 50 mm, Waters). The flow rate was 0.6 mL min^{-1} and the gradient was as follows: 95% Solvent A (0.1% formic acid in water) with 5% solvent B (0.1% formic acid in acetonitrile) was held constant for 0.5 min, followed by a linear gradient to 100% B over the next 2.1 min. After 1 min at 100% solvent B, the gradient was returned to 95% solvent A and 5% solvent B over 0.2 min. The ESI capillary voltage was 3 kV, cone voltage 30 V and collision energy 4 eV. The MS acquisition rate was 10 spectra per second and m/z data ranging from 50 to 2000 Da was collected. Mass accuracy was achieved using a reference lock mass scan, once every 10 s. Water was removed by lyophilisation using a FreeZone Benchtop Freeze Dry System. The purity of the peptides used in the biological assays were determined to be greater than 95% in all instances.

1D-NMR based Real-time Enzymatic Assay

NMR sample preparation

The CoREST ternary complex was purified and used in this assay. The protein was gel filtrated in buffer containing 10 mM Tris pH 7.5, 50 mM KCl and 0.5 mM TCEP. Substrate peptides were dissolved in the same buffer as the gel filtration buffer.

NMR experiments

Substrate peptide samples were prepared to yield a final concentration in a range from 50 μM to 800 μM (final volume of 600 μl, in a 5 mm Wilmad®NMR tube) with 5% D_2O. A reference ^{1}H 1D spectrum was recorded using excitation sculpting water suppression, 4 dummy scans, 16 scans, a recovery delay of 1 s and an acquisition time of 2.04 s, leading to a total acquisition time of 61 s. 16k complex points were recorded with a sweep width of 8012 Hz. Protein was added to start the enzymatic reaction with a final concentration of 50 – 200 nM in a final volume of 600 μl. Inhibitors were pre-incubated with the complex for 30 minutes. One round of gradient shimming was performed followed by the acquisition of a series of 1D ^{1}H NMR spectra with the same parameters as the reference spectrum. The time between the addition of the CoREST complex and the acquisition of the first 1D 1H NMR experiment was started was approximately 80 s and this 'deadtime' was noted for each time series. 1D-NMR spectra were recorded on a Bruker Avance 500 over a certain time course.

Analysis of NMR spectra

The NMR spectra were processed using NMRPipe (Delaglio et al., 1995), using a 1.8 Hz exponential line broadening prior to Fourier transformation. A polynomial baseline correction as implemented in NMRPipe was applied after the Fourier transform. All 1D ^{1}H NMR spectra within a time series were collected into a pseudo 2D spectrum. Signal intensities were determined using the program FuDA as described previously (Hansen et al., 2007) by fitting a mixed Gaussian/ Lorentzian line shape to each peak and assuming a common line shape for a given peak during a time series (i.e., line shape and peak positions are independent of time). Subsequently the peak intensities were converted to concentrations (in μM) by using a reference sample with known concentration.

Data analysis of NMR based enzymatic assay

Least-squares fitting of kinetic parameters: A program was written in C++ to analyze the obtained substrate concentrations versus time and to extract kinetic parameters and standard errors. In the program the ordinary differential equations that describe the kinetics were integrated numerically over the time domain using a controlled adaptive Runge-Kutta Cash-Karp (Bader and Deuflhard, 1983; Abramowitz and Stegun, 1965) stepper in the odeint function implemented in the C++ boost class library (https://www.boost.org). Thus, for each set of rate constants, $\{k_j\}_j$, and initial substrate concentrations $[S]_{0,i}$, the progression curves originating for different initial substrate concentration but same rates constants, $[S]_{calc}(t;\ S_{0,i},\ k_j)$ were calculated.

Best-fit parameters were obtained using a Levenberg-Marquardt least-squares fitting algorithm similar to that described by Press et al. (1992). The model fitting parameters included the initial concentrations of the substrate and the micro-kinetic rate constants, expect for the second-order substrate-enzyme association rate, k_1. Specifically, the χ^2 that was minimized in the fit was defined as

$$\chi^2(t; S_{0,i}, k_j) = \sum_{i,k} \frac{\left([S]_{calc}(t_k; S_{0,i}, k_j) - [S]_{i,exp}(t_k)\right)^2}{\sigma^2}$$

where the sum over i is the experiments with different initial substrate concentration and the sum over k is the different time points, where the substrate concentration was observed experimentally. The uncertainty σ was set to 1 μM for all points as judged from the signal-to-noise. The derivatives $d[S]_{calc}/dS_{0,i}$ and $d[S]_{calc}/dk_j$ used to generate the Jacobi matrix for the least-squares fit were obtained numerically and only elements of the Jacobi matrix that were know *a priori* to be non-zero were calculated.

Extensive grid searches were performed initially to obtain good starting parameters for the fit. Standard errors of the obtained model parameters were determined from the co-variance matrix (Press et al., 1992) or by a bootstrap approach (500 runs) (Efron and Tibshirani, 1986). Each least-squares fit of the singly modified substrates K4MeK9 and K4K9Ac took approximately 15 min on a desktop computer with sixteen 3.2 GHz processors. Significance p-levels were calculated using F-tests and the incomplete beta-function. The program and source codes are available from the authors upon request.

Considerations made in regards to the least-squared analysis: It was assumed that the second order association rate constant between E and S and between E* and S are identical and equals k_1. Initial analyses, grid searches, and chi-square analyses showed that the data does not contain sufficient information for an accurate value of k_1 to be determined, yet k_1 is close to the diffusion limit. In all the analyses it was therefore assumed that k_1 = 200,000 $s^{-1}M^{-1}$. Larger values for k_1 increased the time for solving the coupled differential equations, however it did not improve the chi-square, while smaller values slightly increased the obtained chi-square, χ^2.

The reaction scheme in Figure 3 is circular between the four species E, ES, E*S and E*. Therefore, although there are eight rate constants between E, E*, ES and E*S, there are only seven independent rates if the system is to be thermodynamically stable. In the analysis, the value for the disassociation of E*S was chosen to be calculated from the other rate constants:

$$k_{-1*} = \frac{k_{-ES}}{k_{ES}} \frac{k_E}{k_{-E}} k_{-1} = \frac{K_{eq}(E)}{K_{eq}(ES)} k_{-1}$$

Expression for reaction rates at short times: As discussed in the main text, since $k_E + k_{-E}, k_{ES} + k_{-ES} \ll k_{cat,E}, k_{-1}$ two limiting cases are considered. For short times, $t \ll 1/(k_E+k_{-E}), 1/(k_{ES}+k_{-ES})$ the two forms of the enzyme E and E* present at the start of the reaction are effectively converting the substrate independently. It is assumed that the equilibrium between E and E* has been reached by the start of the experiment and it not perturbed initially, thus the population of E, p_E, immediately before adding substrate is given by,

$$K_{eq}(E) + 1 = \frac{[E^*]}{[E]} + \frac{[E]}{[E]} = \frac{1}{p_E}$$

The overall rate is given by

$$v_0 = -\frac{dS}{dt} = [ES]k_{cat,E} + [E^*S]k_{cat,E*}$$

Assuming steady state for both ES and E*S, that is, d[ES]/dt = 0 and d[E*S]/dt = 0 gives:

$$v_0 = \frac{E_0}{1 + K_{eq}(E)} \frac{k_{cat,E} + k_{cat,E*}K_{eq}(E)}{1 + K_{M,E}/[S] + K_{M,E*}/[S] + K_{M,E}K_{M,E*}/[S]^2}$$

where

$$K_{M,E} = \frac{k_{cat,E} + k_{-1}}{k_1} \qquad K_{M,E*} = \frac{k_{cat,E*} + k_{-1}K_{eq}(E)/K_{eq}(ES)}{k_1}$$

It is noted that for short times the initial rate v_0 cannot be cast in a form of apparent Michaelis-Menten parameters. Still, when the kinetic parameters and initial substrate concentration are known, the limiting initial rate can be calculated using the equation above as shown in Figure 2 (dotted lines).

Expression for apparent Michaelis–Menten parameters for long times: For times $t \gg 1/(k_E+k_{-E}), 1/(k_{ES}+k_{-ES})$ an equilibrium between E, E*, ES and E*S is reached and apparent Michaelis-Menten parameters can be calculated. Since equilibrium is assumed to have been reached:

$$K_{eq}(E) = \frac{[E^*]}{[E]} \qquad \text{and} \qquad K_{eq}(ES) = \frac{[E^*S]}{[ES]}$$

The total enzyme concentration, $[E]_0$, is given by:

$$[E]_0 = [E] + [ES] + [E^*] + [E^*S] \qquad \Rightarrow$$

$$[E] = \frac{[E]_0 - [ES](1 + K_{eq}(ES))}{1 + K_{eq}(E)}$$

and assuming steady state for ES and E*S gives:

$$v_0 = \frac{k_{cat,E}[E]_0\left(1 + K_{eq}(ES)\frac{k_{cat,E^*}}{k_{cat,E}}\right)}{1 + K_{eq}(ES) + (1 + K_{eq}(E))\frac{K_{M,E}}{[S]}}$$

where the apparent Michaelis-Menten parameters are:

$$K_{M,app} = K_{M,E}\frac{1 + K_{eq}(E)}{1 + K_{eq}(ES)}$$

$$k_{cat,app} = k_{cat,E}\frac{1 + K_{eq}(ES)\frac{k_{cat,E^*}}{k_{cat,E}}}{1 + K_{eq}(ES)}$$

It should be noted that these apparent parameters are only valid after equilibrium has fully been reached (dashed lines in Figure 2).

Small-Angle Light Scattering (SAXS)

Purified CoREST ternary complex was concentrated to 1 mg/ml before analysis. Data were collected at Diamond Light Source small angle scattering B21 beam line (Didcot, Oxford, UK). The datasets were analyzed using ScÅtter (Hura et al., 2009). A structural model based on the crystal structure of crystal structures of HDAC1:MTA1 (PDB: 4BKX) and LSD1:RCOR1 (PDB: 2V1D) was calculated using CORAL (Petoukhov et al., 2012). The comparisons of theoretical scattering curves and the experimental datasets were performed using CRYSOL (Svergun et al., 1995). Data collection and analysis details are described in supplementary material Table S7.

Chemical cross-linking mass spectrometry

Isotopically-coded NHS-activated ester cross-linker CBDPSS was used to cross-link the protein sample. Purified CoREST complex was buffer exchanged into cross-linking buffer (50 mM HEPES and 50 mM potassium acetate) and then concentrated to 1 mg/ml. Protein was mixed with 0.2-10 mM of CBDPSS in a v/v ratio of 1:1. Reaction was performed at room temperature for 1 hour and stopped with a final concentration of 40 mM $(NH_4)_2CO_3$. Cross-linked sample were analyzed by SDS-PAGE. The gel bands corresponding to the cross-linked complex were cut and analyzed by LC-MS. Cross-linked peptides were identified using the xQuest (Leitner et al., 2014).

Histone expression and purification

The full-length *Xenopus* histones (H2A, H2B, H3(K4C) or H4) in a pET expression vector were expressed in BL21(DE3)pLysS *E. coli* and induced with IPTG (0.2 mM) at 37°C for 3 h. Cells were pelleted and resuspended with histone wash buffer (50 mM Tris pH 7.5, 100 mM NaCl, 1 mM EDTA and 1% Triton X-100) followed by lysed with French press. The inclusion bodies were pelleted, washed with histone wash buffer without Triton X-100, and then resuspended in histone unfolding buffer (6 M guanidinium chloride, 20 mM Tris pH 7.5 and 10 mM DTT) and then buffer exchanged into IEX buffer (7 M urea, 10 mM Tris pH 7.8, 1 mM EDTA and 5 mM BME). The histone was purified by tandem HiTrap Q-SP columns with a NaCl gradient from 0 mM to 500 mM with IEX buffer. After dialysis against water, histone proteins were lyophilized to dryness.

Preparation of propargylamine-containing mimic of dimethyl Lys4 histone H3

The histone H3 (K4C) protein was dissolved in reaction buffer (4 M guanidinium chloride, 1 M HEPES pH 7.8, 10 mM L-Met), and DTT was added to a final concentration of 10 mM. The mixture was heated at 37°C for 1 h and diluted 4-fold with the reaction buffer. Next, 1-methyl-1-(prop-2-ynyl)aziridinium chloride was added to a final concentration of 15 mM in an ice bath. The mixture was kept at 25°C for 20 h. The reaction was monitored by mass spectrometry and the product was purified by reversed phase HPLC with a C4 column.

Histone octamer refolding and nucleosome reconstitution

The core histone proteins H2A, H2B, H3(K4C) and H4 were dissolved in unfolding buffer (7 M guanidine, 20 mM Tris pH 7.5 and 10 mM DTT) and dialyzed against high salt buffer (20 mM Tris pH 7.5, 2.0 M NaCl, 1 mM EDTA and 5 mM BME). The octamer

was purified by size exclusion chromatography with a Superdex 200 column. The 185 bp 601 DNA was amplified by PCR from the 601 DNA template and purified by anion exchange chromatography with a TSKgel column.

The histone octamer and DNA were mixed at a 1:1 molar ratio in high salt buffer (10 mM Tris 7.5, 2.0 M KCl, 1 mM EDTA and 1 mM DTT), and the mixture was gradually dialyzed to low salt buffer (10 mM Tris pH 7.5, 0.25 M KCl, 1 mM EDTA and 1 mM DTT) over 36 h. The nucleosomes were purified by anion exchange chromatography with TSKgel and dialyzed to 20 mM Tris pH 7.5 and 1 mM DTT. Due to the zinc ion in the CoREST complex, we did not add 1 mM EDTA to the nucleosome storage buffer.

Structural determination of the CoREST complex using negative stain EM

The CoREST ternary complex was purified through a Superose 6 column and the peak fraction was concentrated and further purified in a 5%–25% sucrose density gradient (with 0%–0.1% glutaraldehyde). The sucrose density gradient was made in buffer containing 20 mM HEPES pH 7.5 and 40 mM NaCl. The gradient was manually fractionated with a fraction volume of 175 μl. The fraction containing intra-complex cross-linked sample was selected and buffer exchanged into 25 mM Tris pH 7.5, 50 mM potassium acetate and 0.5 mM TCEP.

Negative-stain grids (carbon film 400 mesh copper grid, Agar Scientific) were prepared by glow-discharging in an auto sputter coater (E5200, Quorum Technologies) for 30 s at 10 mA. 50 ng of the CoREST ternary complex was applied onto the grid and the excess liquid was blotted after 1-minute incubation. 2% uranyl acetate was used to stain the sample. The grid was visualized on a JEOL 2010F 200kV electron microscope and micrographs were taken using a Gatan Ultrascan 4000 camera at University of Warwick. 364 micrographs were collected with defocus values of −0.5 μm, −1.0 μm, −1.5 μm, and −2.0 μm. EMAN2 (Tang et al., 2007), Relion2.1 (Scheres, 2012) and Cryosparc (Punjani et al., 2017) were used for data analysis.

Structural determination of the CoREST complex bound to synthetic site-specific nucleosome using negative stain EM

The CoREST complex was purified through Superose 6 column in buffer containing 20 mM HEPES and 50 mM NaCl. A propargylamine-containing mimic of dimethyl Lys4 histone H3 was prepared as previously described (Pilotto et al., 2015). The modified histone H3 and *E. coli* Expressed H2A, H2B and H4 were then assembled with 185 bp 601 nucleosomal DNA as previously reported (Wu et al., 2018). Interactions of the nucleosome with the CoREST complex were analyzed on a 0.7% agarose gel buffered in 0.5x TB (45 mM Tris, 45 mM boric acid). The gel was first stained with ethidium bromide and visualized using UV to analyze the DNA and, the same gel was then stained with InstantBlue to analyze the protein.

For preparing sample for negative stain, the CoREST complex was mixed with nucleosome in a molar ratio of 3:1. The mixture was incubated at room temperature for 2 hours and purified through a Superdex 200 (3.2/300) column with a fractionation size of 50 μl. The sample from each fraction was analyzed on a 0.7% agarose gel in 0.5x TB buffer. The gel was stained with ethidium bromide and visualized using UV. The fraction that contained the highest percentage of nucleosome:LHC in a 1:1 complex was selected, and cross-linked with 0.01% glutaraldehyde. 5 μl of cross-linked sample was applied on glow discharged grid (carbon film 400 mesh copper grid, Agar Scientific). The sample was stained with 2.5% uranyl acetate for 1 min. The grid was visualized using a Talos F200C 200kV electron microscope at the MRC Toxicology Unit (Leicester). 384 micrographs were collected using a Ceta 16M CMOS camera (total electron dose 30-40 e-/Å^2 for 1 s exposure). Micrographs were collected using FEI EPU software with defocus values of −1.0 μm, −1.5 μm, and −2.0 μm. Relion3.0 (Zivanov et al., 2018) was used for data analysis.

Cryo Electron Microscopy sample preparation and imaging

Preparation of cross-linked protein sample

The CoREST ternary complex was gel filtrated in buffer containing 25 mM HEPES and 50 mM potassium chloride. Protein was then concentrated to 0.6 mg/ml or 0.8 mg/ml and mixed with glutaraldehyde (0.15%) or BS^3 (4 mM) in a v/v ratio of 1:1. Reactions were performed at room temperature for 5 minutes and stopped with a final concentration of 50 mM Tris.

Preparation of vitrified specimen

3 μl of 0.03 mg/ml sample was applied on the graphene oxide coated Quantifoil 300 mesh Au R1.2/1.3 grid. The sample was blotted for 4 s after 30 s waiting time (4°C, 100% humidity) with a blot force of 10 and then plunged into liquid ethane (FEI Vitrobot).

Data acquisition and processing

Datasets were collected on Titan Krios microscope operated at 300 kV equipped with a Gatan Quantum energy filter, a Gatan K2 summit direct electron camera (Gatan) and a Volta phase plate (Thermo Fisher Scientific). Movies were taken in EFTEM with a slit width of 20 eV and at a nominal magnification of 105kx corresponding to a calibrated pixel size of 1.4 Å at the specimen level. Each movie comprises 36 sub-frames with a total dose of 32 e-/Å^2, exposure time was 14 s with a dose rate of 4.47 e-/pixel/s on the detector. Data acquisition was done using FEI EPU software at −0.5 μm defocus. For data acquisition without the Volta phase plate, movies were taken at a nominal magnification of 130kx corresponding to a calibrated pixel size of 1.08 Å at the specimen level. Each movie comprises 48 sub-frames with a total dose of 39 e-/Å^2, exposure time was 12 s with a dose rate of 3.857 e-/pixel/s on the detector. Data acquisition was done using FEI EPU software at −3.3 μm, −3.0 μm, −2.7 μm and −2.4 μm defocus. The frame images of each micrograph were aligned and averaged for correction of beam-induced drift using MotionCor2 (Zheng et al., 2017). The local motion within a micrograph was corrected using 5 × 5 patches without dose-weighting. The defocus values of the micrographs were measured by Gctf-v1.06 (Zhang, 2016). SPHIRE-crYOLO was used to pick particles (Wagner et al., 2019). Picked particles were then analyzed and 3D model were generated using Relion3.0 (Zivanov et al., 2018).

QUANTIFICATION AND STATISTICAL ANALYSIS

Experimental Replicates and Quantification

All data are represented as SEM (standard error of the mean). There are 3 experimental replicates.

Statistical Analysis

All data are presented as mean ± SEM and analyzed using Prism (Graphpad). Student's t test was used for single variable comparison between two groups. Data are presented as ± SEM.

P values are shown in the form: *** $p < 0.001$, or **** $p < 0.0001$.

DATA AND CODE AVAILABILITY

The SAXs data is available from SASDB: SASDH45. The EM map of negative stained CoREST complex, cryoEM map of glutaraldehyde crosslinked CoREST complex, cryoEM map of BS^3 crosslinked CoREST complex (open and closed) and negative stain map of the CoREST:nucleosome complex are available from EMDB: EMD-10626, EMDB: EMD-10627, EMDB: EMD-10628, EMDB: EMD-10629, EMDB: EMD-10630. Unique materials are available from the authors.

FuDA is available from: https://www.ucl.ac.uk/hansen-lab/.

Cell Reports, Volume *30*

Supplemental Information

Mechanism of Crosstalk between the LSD1 Demethylase and HDAC1 Deacetylase in the CoREST Complex

Yun Song, Lisbeth Dagil, Louise Fairall, Naomi Robertson, Mingxuan Wu, T.J. Ragan, Christos G. Savva, Almutasem Saleh, Nobuhiro Morone, Micha B.A. Kunze, Andrew G. Jamieson, Philip A. Cole, D. Flemming Hansen, and John W.R. Schwabe

Supplementary Figures and Tables:

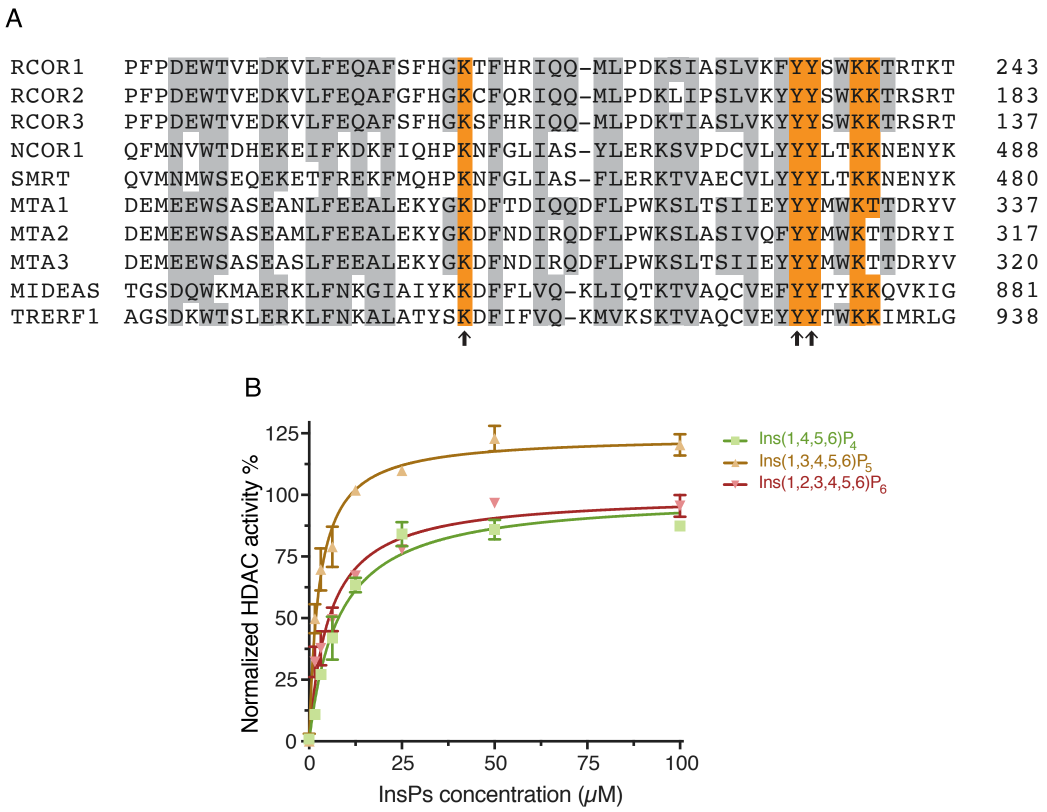

Figure S1. Related to Figure 1. Regulation of the HDAC activity of the CoREST complexes by inositol phosphates.

(A) Sequence alignment of the N-terminal SANT domain of the RCOR1 protein to several other SANT domain containing proteins. Residues that mediate the interaction with Ins(1,4,5,6)P_4 from SMRT SANT domain are highlighted in orange. Black arrows indicate the residues that are essential for the inositol phosphate binding and complex activation.

(B) Activation of HDAC1 in the LSD1:RCOR1:HDAC1 complex by different inositol phosphates. Data were normalized to the HDAC activity in the present of 200 μM of Ins(1,4,5,6)P_4. Error bars indicate the SEM (n=9).

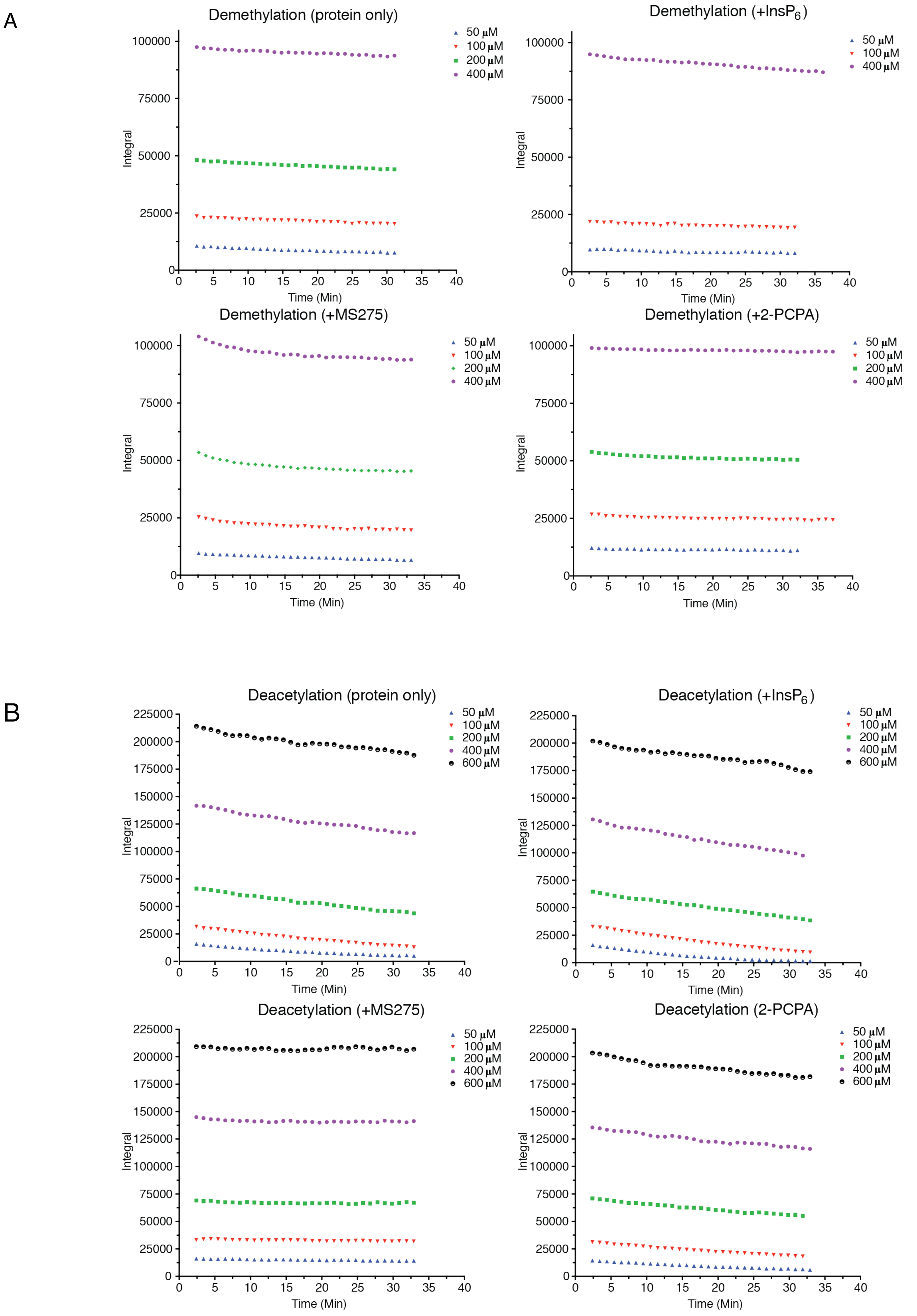

Figure S2. Related to Figure 2.

(A) Time-course data obtained by integrating the substrate peak of monomethylated K4 of the H3 K4meK9 substrate. 200 nM of CoREST ternary complex was used in the experiments.

(B) Time-course data obtained by integrating the substrate peak of acetylated K9 of the H3 K4K9ac substrate. 50 nM of CoREST ternary complex was used in the experiments.

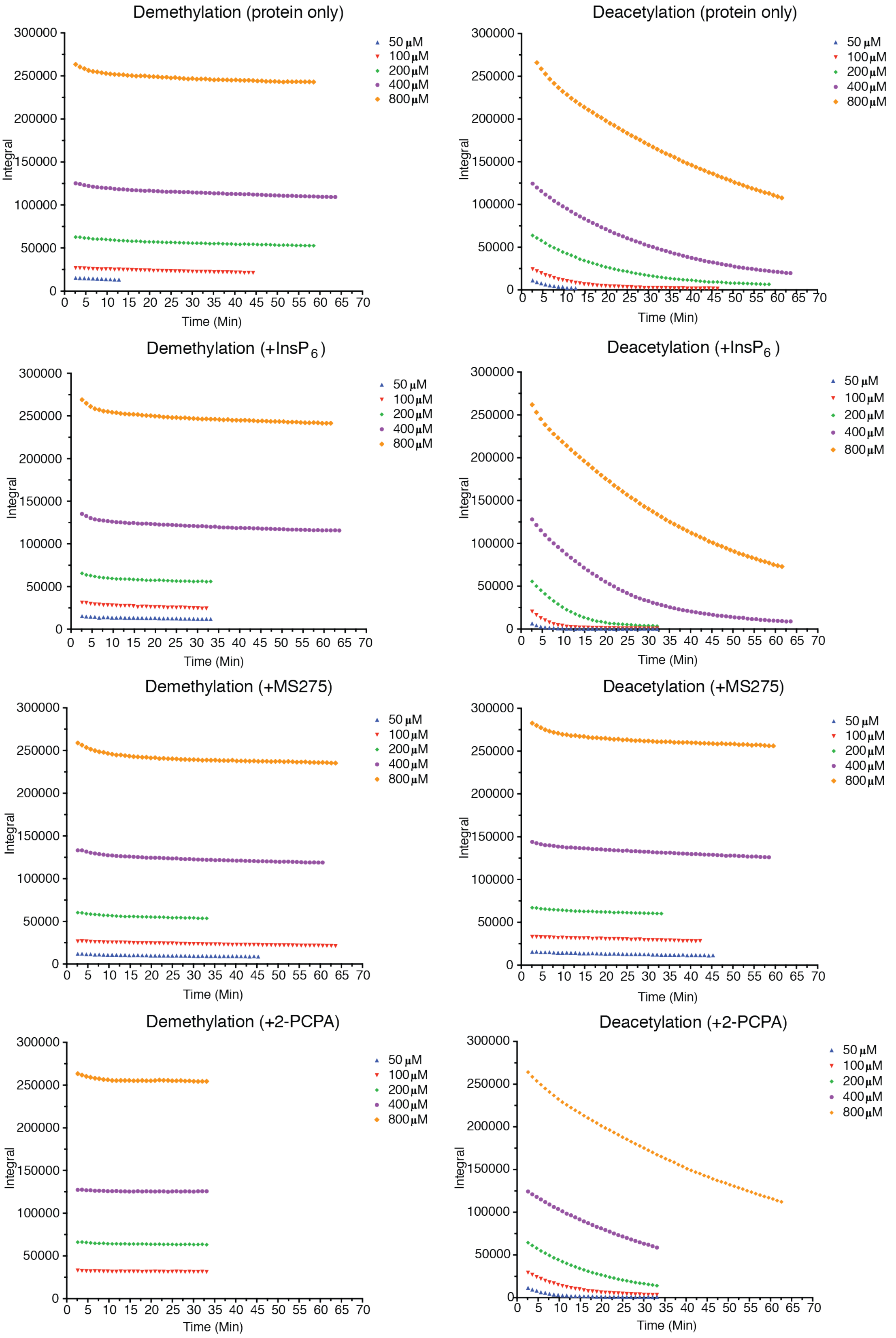

Figure S3. Related to Figure 2. Time-course data obtained by integrating the substrate peak of monomethylated K4 and acetylated K9 of the H3 K4meK9ac substrate. 200 nM of CoREST ternary complex was used in the experiments.

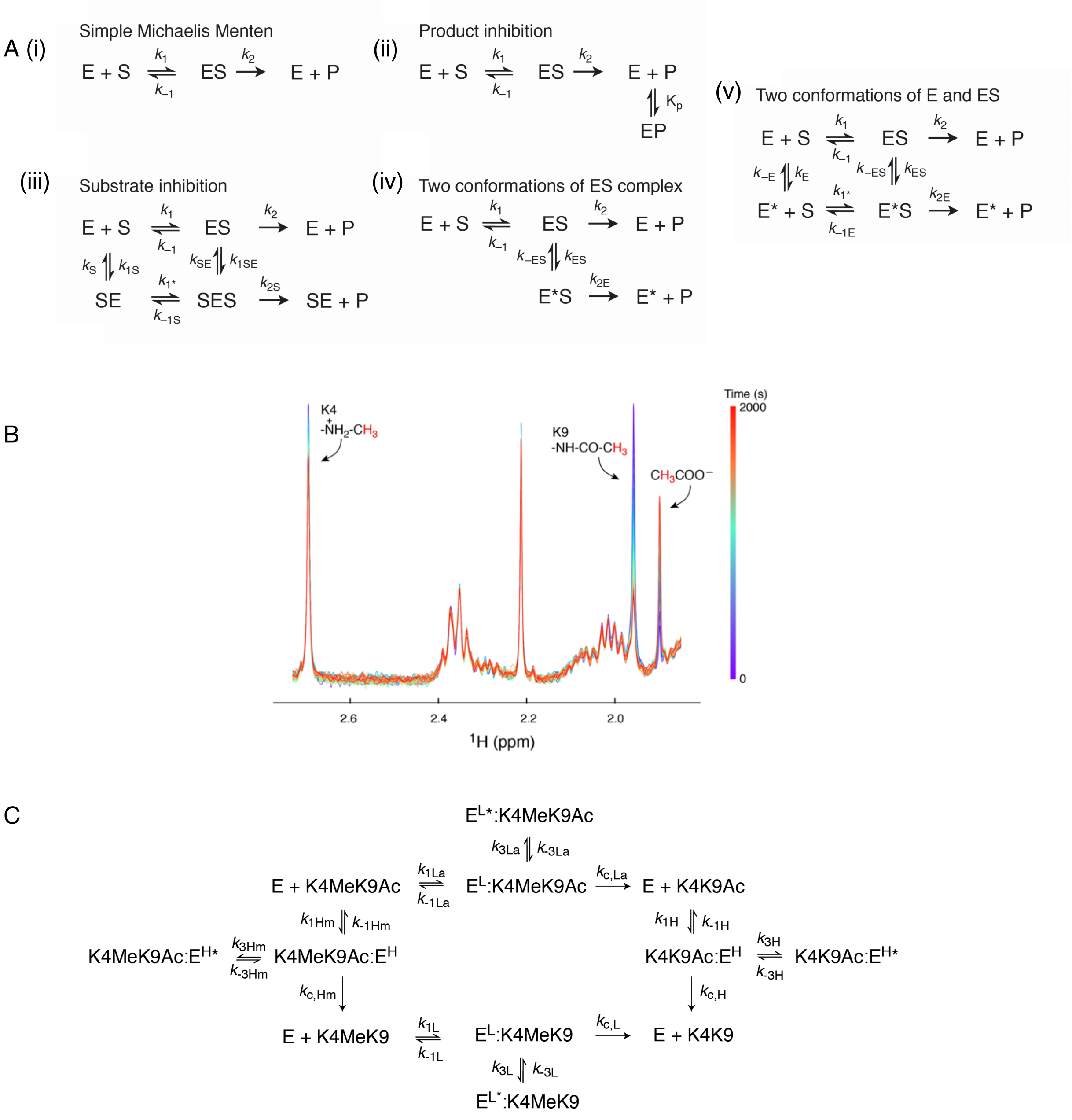

Figure S4. Related to Figure 2.

(A) Models considered for the demethylation of H3 K4meK9 and the deacetylation of H3 K4K9ac: A simple (i) Michaelis-Menten reaction scheme; (ii) Michaelis-Menten reaction with product-inhibition. (The equilibrium between E+P and EP is described by the equilibrium constant, K_p, and is thus assumed to be reached instantaneous); (iii) Michaelis-Menten with generalised substrate inhibition of both the free enzyme, E, and the enzyme-substrate complex, ES; (iv) Reaction scheme where the enzyme-substrate complex can exist in two conformations; (v) Reaction scheme where both the free enzyme, E, and the enzyme-substrate complex, ES, can exist in two conformations. For all reactions k_1 = 0.25×10^6 $M^{-1}s^{-1}$ corresponding to near diffusion limit on-rate. Small changes in k_1 did not change the obtained χ^2. For the reaction schemes in (iii) and (v) $k_{1^*} = k_1$. Also, there are only 7 fitting parameters in scheme (iii) and (v), despite 8 shown, due to the cyclic nature of the schemes. Thus, in (v), k_{-1E}, can be calculated from k_{1^*}, k_{-E}, k_E, k_1, k_{-1}, k_{-ES}, k_{ES}.

(B) Series of ^{1}H NMR spectra showing the demethylation and deacetylation of the substrate H3K4meK9ac (50 μM) by CoREST (200 nM).

(C) Reaction scheme used for the analysis of combined demethylation and deacetylation of the doubly modified histone H3 K4meK9ac substrate. All second-order association rates, k_{1Hm}, k_{1H}, k_{1La}, k_{1L} were fixed to $2\cdot10^5$ $M^{-1}s^{-1}$ as discussed in the main text

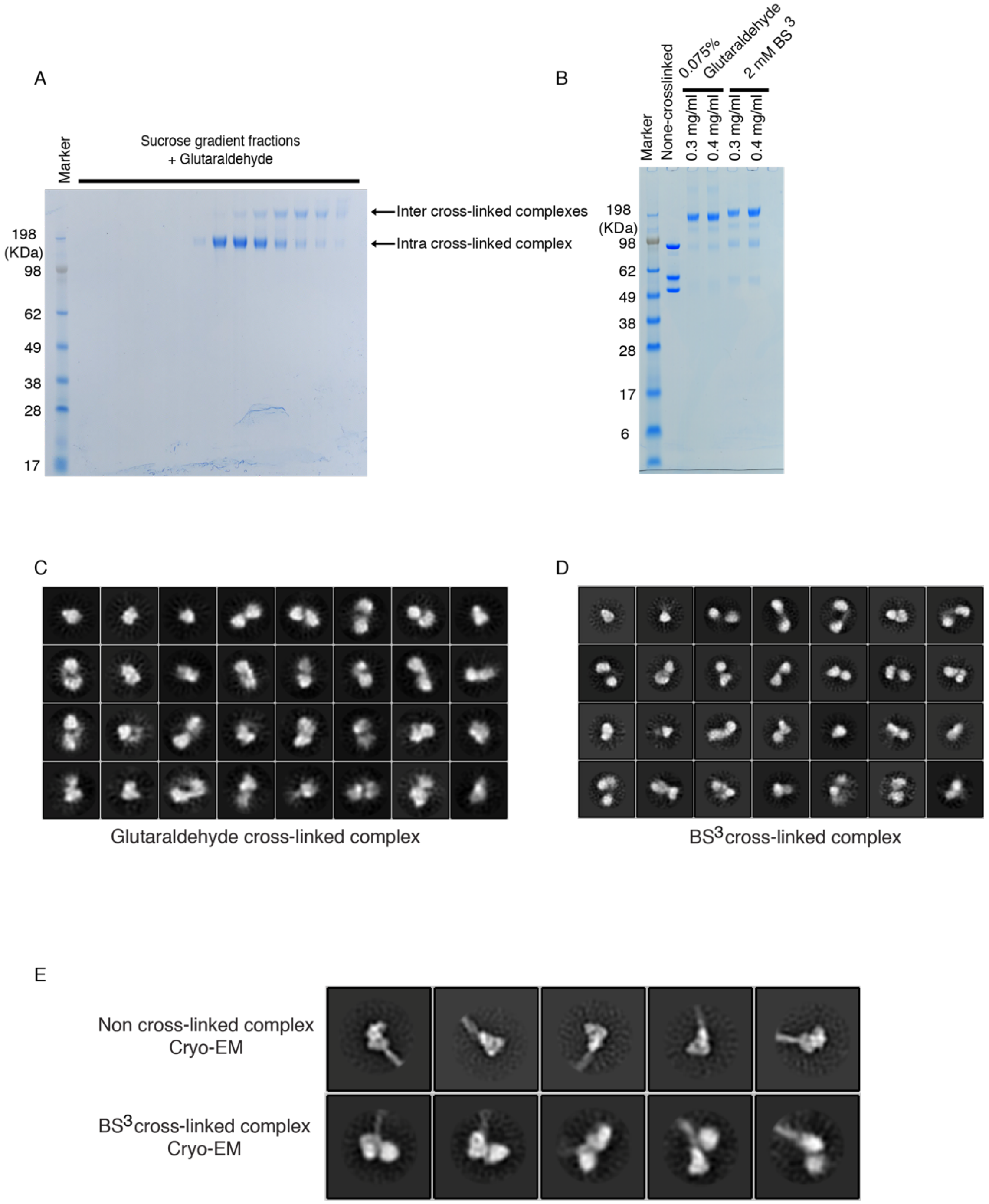

Figure S5. Related to Figure 4. Cryo-electron microscopy of the CoREST complex.

(A) Purification and crosslinking of the CoREST complex using a 5-25% sucrose (+0.1% glutaraldehyde) gradient. NuPAGE gel analysis of fractions 1-14 from sucrose gradient.

(B) Crosslinking the CoREST complex with 0.075% glutaraldehyde and 2 mM BS^3 in a microfuge tube for 5 minutes at room temperature.

(C) Selected 2D-classes of the CoREST complex cross-linked with glutaraldehyde.

(D) Selected 2D-classes of the CoREST complex cross-linked with BS^3.

(E) Selected 2D-class averages of the non cross-linked CoREST complex and BS^3 cross-linked CoREST complex in Cryo-EM.

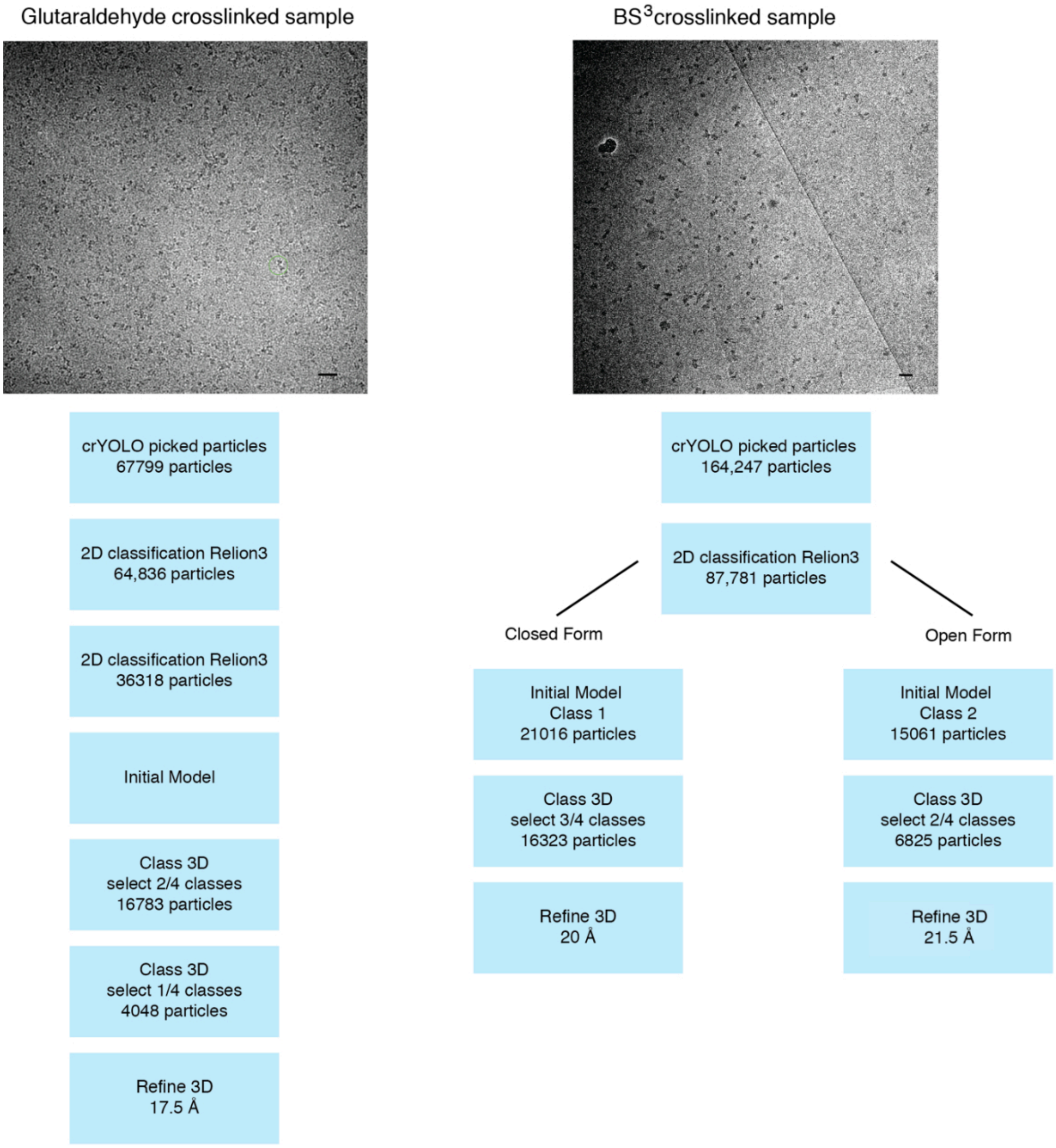

Figure S6. Related to Figure 4. Typical micrographs and flow chart for the Relion 3.0 processing of the cryo-EM datasets for the glutaraldehyde and BS^3 crosslinked CoREST samples. The scale bar in the micrographs is 20 nm.

A

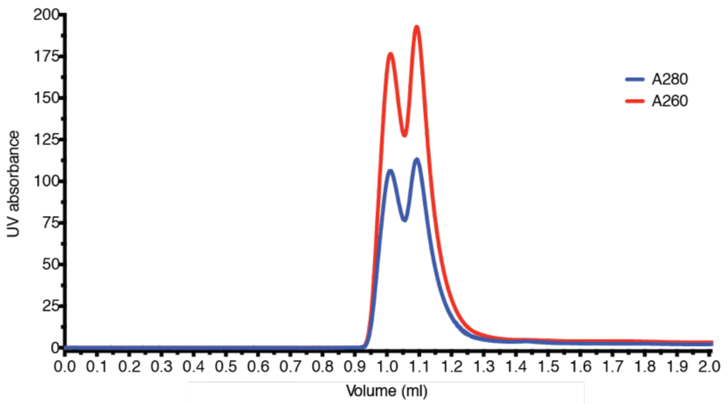

B

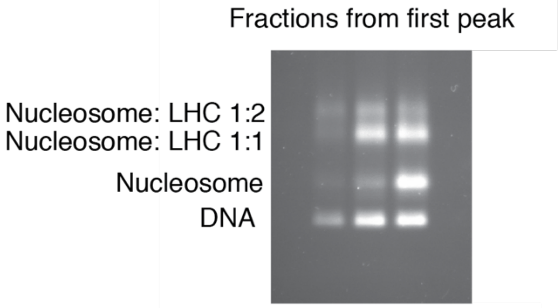

C

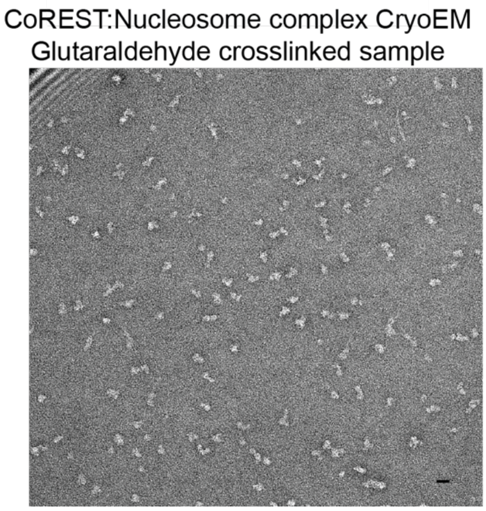

Figure S7. Related to Figure 5. Purification and negative stain microscopy of the nucleosome bound CoREST complex.

(A) CoREST complex was mixed with nucleosome in a molar ratio of 3:1 and then purified through a 2.4 ml Superdex 200 (3.2/300) column with a fraction size of 50 μl.

(B) The fractions from the first peak (0.9ml - 1.05 ml) were analysed on a 0.7% agarose gel in 0.5x TB buffer. The gel was stained with ethidium bromide and visualized by UV.

(C) Typical electron micrograph of the negatively stained CoREST:nucleosome complex. The scale bar is 20 nm.

Table S1. Related to Figure 2. Characterisation data for purified peptides.

Peptide	Calculated MW [M+H]+ (Da)	Run time (min)	ES+ peaks (m/z)	Purity (%)	Yield (%)
**H-ARTK(Me)QTARKSTGGKAPRKQLA-NH_2 (H3(1-21)K4me)**	2267.3530	11.797[a]<br>13.223[b]	1134.5880 $[M+2H]^{2+}$<br>756.7203 $[M+3H]^{3+}$<br>567.8007 $[M+4H]^{4+}$<br>454.4461 $[M+5H]^{5+}$<br>378.6996 $[M+6H]^{6+}$	>99	45
**H-ARTKQTARK(Ac)STGGKAPRKQLA-NH_2 (H3(1-21)K9Ac)**	2295.3441	11.783[a]<br>13.193[b]	1148.6685 $[M+2H]^{2+}$<br>766.1127 $[M+3H]^{3+}$<br>574.8332 $[M+4H]^{4+}$<br>460.0672 $[M+5H]^{5+}$<br>384.1527 $[M+6H]^{6+}$	>99	36
**H-ARTK(Me)QTARK(Ac)STGGKAPRKQLA-NH_2 (H3(1-21)K4meK9ac)**	2309.3636	11.823[a]<br>13.300[b]	1155.7358 $[M+2H]^{2+}$<br>770.8013 $[M+3H]^{3+}$<br>578.3477 $[M+4H]^{4+}$<br>462.8733 $[M+5H]^{5+}$<br>385.7206 $[M+6H]^{6+}$	>99	23

Analytical reverse-phase HPLC using a Dionex Ultimate 3000 system with a Phenomenex Aeris 5 μm Peptide XB-C18 100 Å packed column with dimensions of 150 x 4.6 mm, using the following gradients: (a) 5-100% MeCN/H_2O (0.1% TFA), 15 min gradient and (b) 5-100% MeCN/H_2O (0.1% TFA), 30 min gradient.

Table S2. Related to Figure 2. Assessment of models considered for the demethylation of H3 K4meK9[a)]

Model[a)]	CoREST	$InsP_6$ [b)]	MS275[c)]	Total normalised χ^2 [d)]	p-value [e)] w.r.t. Model (i)	p-value [e)] w.r.t. Model (ii)	p-value [e)] w.r.t. Model (iv)
	χ^2 / χ^2_{red}						
**(i)**	61.7/0.54	51.0/0.60	333/2.94	1251.4			
**(ii)**	49.7/0.44	49.9/0.59	190/1.69	812.9	2.1×10^{-32}		
**(iii)**	60.7/0.56	47.3/0.59	332/3.07	1234.7	0.501	N/A	
**(iv)**	32.7/0.29	48.3/0.58	123/1.11	572.8	7.1×10^{-56}	1.6×10^{-26}	
**(v)**	28.9/0.26	48.4/0.60	37.4/0.34	322.0	1.2×10^{-92}	1.8×10^{-63}	5.0×10^{-40}

a) The models considered are shown in Figure S4A. b) The CoREST complex was pre-equilibrated with 100 μM $InsP_6$. (c) The CoREST complex was pre-equilibrated with 5 μM MS275 HDAC inhibitor. (d) The sum of the χ^2 calculated for CoREST only, CoREST with $InsP_6$, and CoREST with MS275. Furthermore, the χ^2 was normalised as described by (Press et. al., 1992). (e) The probability value (significance), p-value, was calculated using F-test (Press et al., 1992). Model (v) is the most significant of the models considered.

Table S3. Related to Figure 2. Assessment of models considered for the deacetylation of H3 K4K9ac[a)]

Model[a)]	CoREST	$InsP_6$ [b)]	2-PCPA[c)]	Total normalised χ^2 [d)]	p-value [e)] w.r.t. model (i)	p-value [e)] w.r.t. model (ii)	p-value [e)] w.r.t. model (iv)
	χ^2 / χ^2_{red}						
**(i)**	1203/8.1	1675/12	1014/7.3	752.6			
**(ii)**	1167/7.9	1575/11	760/5.5	677.1	6.6×10^{-12}		
**(iii)**	1012/7.1	1666/12	963/7.2	704.1	1.8×10^{-5}	N/A	
**(iv)**	691/4.7	1391/9.9	519/3.8	502.9	1.1×10^{-39}	1.4×10^{-30}	
**(v)**	512/3.6	1317/9.6	462/3.5	443.0	9.5×10^{-50}	1.6×10^{-40}	6.3×10^{-13}

a) The models considered are shown in Figure S4A. b) The CoREST complex (50 nM) was pre-equilibrated with 100 μM $InsP_6$. c) The CoREST complex was pre-equilibrated with 5 μM MS275 HDAC inhibitor. d) The sum of the χ^2 calculated for CoREST only, CoREST with $InsP_6$, and CoREST with MS275. Furthermore, the χ^2 was normalised as described by by (Press et. al., 1992). e) The probability value (significance), p-value, was calculated using F-test by (Press et. al., 1992). Model (v) is the most significant of the models considered.

Table S4. Related to Table 1. Full list of kinetic parameters obtained for the demethylation of H3 K4meK9 substrate by CoREST.

	Protein only	$InsP_6$ a)	MS275 b)	2-PCPA c)
χ^2 / χ_{red}^2	28.8 / 0.26	48.2 / 0.60	37.6 / 0.35	43.7 / 0.43
$K_{m,E}$ (μM)	30 ± 21	2390 ± 230	550± 300	394 ± 81
$k_{cat,E}$ (s^{-1})	2.5 ± 1.0	4.61 ± 0.15	3.4 ± 0.9	0.97 ± 0.05
$k_{cat,E}$ / $K_{m,E}$($s^{-1}\mu M^{-1}$)	0.083 ± 0.047	0.0019 ± 0.0002	0.0070 ± 0.0022	0.0025 ± 0.0002
$K_{m,E*}$ (μM)	1.6 ± 1.4	146 ± 25	5.4 ± 1.1	23 ± 5
$K_{m,post}$ (μM)	2.9 ± 1.7	193 ± 30	9.3 ± 1.6	25.7 ± 5.5
$k_{cat,post}$ (s^{-1})	0.032 ± 0.002	0.093 ± 0.007	0.023 ± 0.003	0.0077 ± 0.0012
K_{eq}(E)	7.0 ± 2.4	3.0 ± 0.3	1.7 ± 0.6	7.4 ± 0.5
k_{ex}(E) (s^{-1})	0.0074 ± 0.0024	0.0015 ± 0.0013	0.0028 ± 0.0015	$< 5 \times 10^{-4}$
K_{eq}(ES)	76 ± 28	49 ± 4	146 ± 38	129 ± 30
k_{ex}(ES) (s^{-1})	0.0055 ± 0.0012	0.047 ± 0.020	0.0060 ± 0.0024	0.0050 ± 0.0008

(a) The CoREST complex was pre-equilibrated with 100 μM $InsP_6$. (b) The CoREST complex was pre-equilibrated with 5 μM MS275 HDAC inhibitor. (c) The CoREST complex was pre-equilibrated with 100 μM 2-PCPA LSD1 inhibitor.

Table S5. Related to Table 2. Full list of kinetic parameters obtained for the deacetylation of H3K9ac substrate by CoREST.

	**Protein only**	**$InsP_6$[a]**	**2-PCPA[b]**
**χ^2 / χ_{red}^2**	513 / 3.6	1300 / 9.6	462 / 3.5
**$K_{m,E}$ (μM)**	10570 ± 5700	10640 ± 6700	1190 ± 960
**$k_{cat,E}$ (s^{-1})**	440 ± 290	510 ± 350	45 ± 33
**K_{m,E^*} (μM)**	24 ± 5	5.7 ± 4.2	16 ± 3
**k_{cat,E^*} (s^{-1})**	0.52 ± 0.03	0.4 ± 0.2	0.33 ± 0.06
**$K_{m,post}$ (μM)**	33 ± 6	12 ± 5	23 ± 4
**$k_{cat,post}$ (s^{-1})**	0.70 ± 0.03	0.78 ± 0.18	0.52 ± 0.05
**K_{eq}(E)**	6.8 ± 2.3	1.9 ± 2.1	5.5 ± 2.2
**k_{ex}(E) (s^{-1})**	$< 10^{-7}$	0.14 ± 0.17	0.0031 ± 0.0017
**K_{eq}(ES)**	2560 ± 1620	1990 ± 1600	280 ± 220
**k_{ex}(ES) (s^{-1})**	0.077 ± 0.042	0.094 ± 0.066	0.0028 ± 0.0016

(a) The CoREST complex was pre-equilibrated with 100 μM $InsP_6$. (b) The CoREST complex was pre-equilibrated with 100 μM 2-PCPA LSD1 inhibitor.

Table S6. Related to Figure 2. Combined analyses of K4meK9, K4K9ac and K4meK9ac using time points up to 2000 s.

**Model no.**	**Comments**	χ^2 / χ_{red}^2
1 [a]	*Completely uncoupled reactions:* It is assumed that the HDAC and LSD1 are completely uncoupled, that both demethylation and deacetylation reactions follow Michaelis-Menten and that (*a*) demethylation of K4meK9ac follows the same parameters as K4meK9 (*b*) deacetylation of K4meK9ac follows the same parameters as K4K9ac.	10706 / 20.5
2	*Semi-uncoupled reactions:* It is assumed that the HDAC and LSD1 functions independently, but that there is a substrate dependence. Thus, different Michaelis-Menten parameters are assumed for demethylation of K4meK9ac and K4meK9 and different Michaelis-Menten parameters are assumed for deacetylation of K4meK9ac and K4K9ac.	7147 / 13.8
3	*Semi-coupled reactions:* It is assumed that CoREST can only bind one substrate at once. Thus, if substrate is bound to LSD1 then substrate cannot bind to HDAC. Michaelis-Menten parameters are independent on substrate.	12991 / 24.9
4 [a]	*Coupled reactions:* It is assumed that CoREST can only bind one substrate at once. Substrate dependent Michaelis-Menten parameters are assumed.	2570 / 4.96
5	*Semi-uncoupled reactions with product inhibition.* Same as model 2 but with product inhibition, assuming different dissociation constants, K_i, for (*a*) LSD1 inhibition by K4K9ac (*b*) LSD1 inhibition by K4K9 (*c*) HDAC inhibition by K4meK9 and (*d*) HDAC inhibition by K4K9.	3111 / 6.05
6	*Semi-coupled reactions with product inhibition.* Same as Model 3 but with product inhibition as in Model 5.	3731 / 7.19
7	*Coupled reactions with product inhibition.* Same as Model 4, but with product inhibition.	2529 / 4.91
8 [a]	*Coupled reaction with alternate state of enzyme-substrate complexes (Figure S6).* Assuming $k_{\pm 3L} = k_{\pm 3La} = k_{\pm 3H} = k_{\pm 3Hm}$	1981 / 3.84
9 [a]	*Coupled reaction with alternate state of enzyme-substrate complexes (Figure S6).* Assuming all $k_{\pm 3L} = k_{\pm 3La}$, $k_{\pm 3H} = k_{\pm 3Hm}$	1614 / 3.14
10 [a]	*Coupled reaction with alternate state of enzyme-substrate complexes (Figure S6).*	1589 / 3.10
11 [a]	*Coupled reaction with alternate state of the enzyme-substrate complexes and product inhibition.*	1587 / 3.12
12	*Coupled reaction with alternate state of the free enzyme and enzyme-substrate complexes*	Not converged.

(a) The following *p*-levels are obtained from comparisons of the chi-squared, χ^2, and the degrees of freedoms of the fits. Model 1 *v.s.* Model 4: *p*-level $<10^{-50}$, Model 2 *v.s.* Model 4: Model 4 *v.s.* Model 8: *p*-level = 7×10^{-30}, Model 9 *v.s.* Model 8: *p*-level = 1×10^{-23}, Model 10 *v.s.* Model 9: *p*-level = 0.018, Model 11 *v.s.* Model 10: *p*-level = 0.89. The most significant model is Model 10, that is, a coupled reaction with different rate constants for the different substrates and an exchange of the enzymes with alternate states.

Table S7. Related to Figure 3. Data collection and structure statistics for small angle X-ray scattering analysis

**Sample properties**	
Organism	Homo sapiens
Solvent	25 mM Tris pH 7.5, 50 mM potassium acetate and 0.5 mM TCEP
Components	RCOR1 (86-485)<br>HDAC1<br>LSD1
**Data collection parameters**	
Instructment	Diamond light source B21 (Didcot, UK)
Beam geometry (mm^2)	0.8 × 2 mm
Wavelength (Å)	~ 1
s range ($Å^{-1}$)	0.0032 - 0.38
Exposure time (s)	5
Temperature (K)	298
**Structural parameters**	
Rg (Å) (from P(r))	58.14
Rg (Å) (from Guinier plot)	60
I(0) (from P(r))	0.00682
I(0) (from Guinier plot)	0.00825
Dmax (Å)	158
Porod volume estimate ($Å^3$)	437000
**Molecular mass determination (kDa)**	
From Porod volume	257
From SAXS MoW	265
Calculated molecular mass from sequence	194
**Software employed**	
Primary data reduction	ScÅtter
Data processing	ScÅtter
Ab initio analysis	ScÅtter/DAMIF
Validation and averaging	ScÅtter/DAMAVER
Atomic structure modelling	CORAL
Computation of model intensities and fitting with experimental data	CRYSOL
3D graphics representations	Pymol
